# Dissecting a heterotic gene through GradedPool-Seq mapping informs a rice-improvement strategy

**DOI:** 10.1038/s41467-019-11017-y

**Published:** 2019-07-05

**Authors:** Changsheng Wang, Shican Tang, Qilin Zhan, Qingqing Hou, Yan Zhao, Qiang Zhao, Qi Feng, Congcong Zhou, Danfeng Lyu, Lingling Cui, Yan Li, Jiashun Miao, Chuanrang Zhu, Yiqi Lu, Yongchun Wang, Ziqun Wang, Jingjie Zhu, Yingying Shangguan, Junyi Gong, Shihua Yang, Wuqi Wang, Jianfu Zhang, Huaan Xie, Xuehui Huang, Bin Han

**Affiliations:** 1 National Center for Gene Research, CAS Center for Excellence in Molecular Plant Sciences, Institute of Plant Physiology and Ecology, Shanghai Institutes for Biological Sciences, Chinese Academy of Sciences, Shanghai, 200233 China; 2 University of Chinese Academy of Sciences, Beijing, 100049 China; 3 State Key Laboratory of Rice Biology, China National Rice Research Institute, Chinese Academy of Agricultural Sciences, Hangzhou, 310006 China; 4 Rice Research Institute, Fujian Academy of Agricultural Sciences, Fuzhou, 350018 China; 5 College of Life Sciences, Shanghai Normal University, Shanghai, 200234 China

**Keywords:** Agricultural genetics, Genetic hybridization, Plant breeding, Plant hybridization

## Abstract

Hybrid rice breeding for exploiting hybrid vigor, heterosis, has greatly increased grain yield. However, the heterosis-related genes associated with rice grain production remain largely unknown, partly because comprehensive mapping of heterosis-related traits is still labor-intensive and time-consuming. Here, we present a quantitative trait locus (QTL) mapping method, GradedPool-Seq, for rapidly mapping QTLs by whole-genome sequencing of graded-pool samples from F_2_ progeny via bulked-segregant analysis. We implement this method and map-based cloning to dissect the heterotic QTL *GW3p6* from the female line. We then generate the near isogenic line NIL-FH676::*GW3p6* by introgressing the *GW3p6* allele from the female line Guangzhan63-4S into the male inbred line Fuhui676. The NIL-FH676::*GW3p6* exhibits grain yield highly increased compared to Fuhui676. This study demonstrates that it may be possible to achieve a high level of grain production in inbred rice lines without the need to construct hybrids.

## Abstract (web summary)

Developing hybrid rice cultivars requires time consuming random crossing. Here, the authors develop a new next generation sequencing-based quantitative trait locus mapping method to dissect heterotic gene *OsMADS1* and demonstrate the feasibility of pyramiding two genes to achieve large heterotic effect.

## Introduction

Rice heterosis, or hybrid vigor, refers to the increased yield in a hybrid offspring compared to its inbred parental lines. The rice hybrid varieties typically display a grain yield advantage of 10–30% over their parents^1^. Beginning with the first commercial hybrid maize varieties in the 1930s^2^, and the development of hybrid rice in the early 1970s in China^3^, exploitation of heterosis in crop plants has achieved remarkable yield advantages over traditional breeding of inbred lines. To date, hybrid breeding that combines superior alleles from both parental lines to generate a better F_1_ variety is still one of the fastest and most efficient approaches in the breeding of rice as well as in many other crops. However, modern hybrid breeding relying on random crosses between diverse varieties and comprehensive phenotypic selection is still labor-intensive and time-consuming.

In the efforts to uncover the genetic basis of heterosis^4,5^, there are several non-mutually exclusive hypotheses for heterosis, i.e., dominance, overdominance and epistasis^6,7^, with evidences from many molecular genetic experiments that have been performed^8–14^. In recent work, the genetic basis of heterosis with regard to rice grain yield has been explored by an integrated genomic approach to construct a genome map of 1495 elite hybrid rice varieties and the in-depth genetic analyses on 17 F_2_ populations^15,16^. The results showed that a small number of genomic loci from female parents explained a large proportion of the yield advantage of hybrids over their male parents of elite inbred varieties. For most of the heterosis-related loci identified, dominance or incomplete dominance of heterozygous loci plays an important role. Therefore, by optimizing cross design, characterization and dissection of heterotic genes and their allelic distribution in diverse germplasms will greatly enhance genetic improvement of rice. Several important high-yielding or heterosis-related genes have been characterized, such as the *Ghd7* gene^17^ from the male parent (restore line) MH63 and the *Ghd8* gene^18,19^ from the male lines HR5 and 9311. In contrast, yield-associated heterotic genes from female parents (male-sterile lines) remain largely unknown in rice. Genetic mapping for complex agronomic traits through linkage analysis or genome-wide association study (GWAS) showed its great power in the last few decades^20–25^, which will help to identify the heterotic genes from female parents in hybrid rice.

In this study, to accelerate the genetic mapping processes, we develop a quantitative trait locus (QTL) mapping approach, GradedPool-Seq (GPS), that combines high-throughput sequencing with bulked-segregant analysis (BSA). This method is to score and assign F_2_ generations derived from a distant cross of parental lines exhibiting contrasting phenotypes into three or more graded groups based on their measured phenotypic values. Compared to previous methods using BSA coupled with whole-genome sequencing, such as MutMap^26^, SHOREmap^27^, next-generation mapping^28^ and QTL-seq^29,30^, the GPS approach has the advantage of performing genetic mapping to simultaneously detect several QTLs at high resolution (~400-kb) by only requiring F_2_ population. Furthermore, we can assess multiple phenotypic traits using one F_2_ population. This method also allows us to rapidly identify heterotic genes. Benefitting from the robust GPS method coupled with follow-up experiments, we identified and validated a heterotic gene, *GW3p6 (OsMADS1)*, from the female line (male-sterile line), that contributed greatly to 1000-grain weight and grain yield per plant in an elite hybrid rice variety Guang-Liang-You-676 (GLY-676). Notably, the near-isogenic line (NIL) NIL-FH676::*GW3p6* produced by introgressing the *GW3p6* allele from the female line (Guangzhan63-4S, hereafter as GZ) into the male line (Fuhui676, hereafter as FH) exhibit grain yield highly increased compared to FH plants. Rice hybrid breeding is currently hindered by bottlenecks of inefficiency and directionlessness^31^, and the results of this study inform that it can open the door to achieving a high level of grain production using inbred lines instead of generating hybrids.

## Results

### Development of the GPS method

**Fig. 1:**
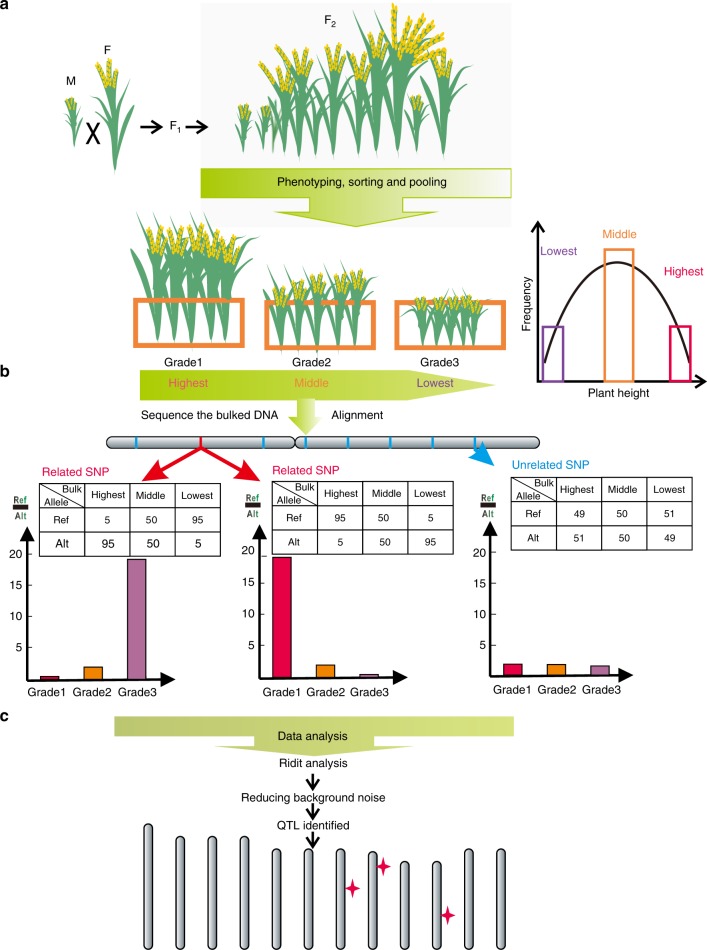
The procedure of the GPS method. **a** F_2_ populations were generated by crossing two parent lines with distinct background and showed vastly different in plant height, followed by the self-pollination of F_1_ generation. Through the observation of plant height, F_2_ populations were classified from the highest to lowest bulk according to their frequency distribution. The letters M and F indicated the male parents and female parents, respectively. **b** The sequence reads of each bulk were aligned against the reference genome sequence to estimate allelic frequencies. We assumed that the depth at each SNP was 100×. If a variance was not related to the phenotype, it would present 50% reference reads and 50% alternate reads in all three bulks (right), whereas the related SNP would have great distinction of the ratio of reference reads to alternate reads among three bulks (left and middle). **c** Data analysis included Ridit analysis and reducing background noise approach. Several associated genetic intervals were mapped and indicated as red four-pointed star

To rapidly identify QTLs for complex traits, we developed a method to map QTLs by directly sequencing graded-pool samples (GPS) from F_2_ progeny using modified BSA. The GPS procedure, including data obtainment, the filtering process, and statistical analysis, can be explained using plant height phenotype as an example (Fig. 1, Supplementary Fig. 1). To begin with, the mapping populations were developed from a cross between two genetically distant parental lines, one with a higher and the other with a lower phenotypic value of plant height, followed by subsequent self-pollination. Given that the parental lines display contrasting target phenotypes controlled by multiple genes, we predicted that the phenotypic variations in plant height among the F_2_ progeny will follow a pattern close to the normal distribution. By assessing plant height in each individual, we classified the F_2_ population into several graded bulks (three graded bulks in the graphic example) from the highest to lowest bulk according to their frequency distribution, as opposed to the two phenotypic tails used in BSA (Fig. 1a). We selected 100 to 150 individuals from each bulk in equal mixed DNA samples with sufficient genomic coverage (average 0.5×–1× per individual) for sequencing to guarantee sequencing accuracy; selection of 20–30% of the total individuals as a bulk is recommended. Next, the sequence reads of each bulk were aligned against the reference genome sequence to estimate allelic frequencies. We assumed that the depth at each single nucleotide polymorphism (SNP) was 100×. If a variance is not related to the phenotype, it would present 50% reference reads and 50% alternate reads in all three bulks; related SNPs would show great distinction in reference reads to alternate reads between the Grade 1 and Grade 3 bulks (Fig. 1b).

Data analysis was carried out as presented in Fig. 1c. We performed the statistical test, Ridit analysis, which adequately examines and analyses ordinal data^32^, for each variant to compute its *p* value. Theoretically, if a variant is closely linked to a phenotype-related gene, its *p* value will be relatively small; however, it is not enough to predict the causative variant due to a large quantity of background noise in SNP calling by the distantly genetic cross. Consequently, background noise reduction must be taken into consideration. The noise-reduction algorithm we implemented in this study was a non-overlap sliding window approach, calculating the ratio of the number of statistically significant variants beyond the set threshold to the total number of variants in a defined genomic interval (~400 kb). Thus, we sought the interval with the largest ratio, i.e., SNPs clusters with significant *p* values in the highest proportion. The relationship between the ratio and chromosomal intervals reveals those genomic regions where QTLs associated with plant height are most likely to be located.

To assess its efficiency and robustness, we applied the GPS pipeline to identify QTLs underlying four agricultural traits (heading date, plant height, flag leaf angle and tiller angle). Multiple QTLs related to these four traits were mapped by GPS, and we focused on the phenotype of plant height. Three F_2_ populations of ~400 rice individuals were generated by crossing parental lines exhibiting a distinct plant height phenotype. According to the phenotypic value, we categorized the F_2_ lines into several ordinal classes from highest to lowest (Supplementary Table 1), followed with sequencing of the pools. After aligning the sequence reads to the reference sequence (IRGSP build 4) via BWA software^33^ and variant calling using GATK^34^, we obtained genome-wide SNP information to conduct filtering procedure step by step, which filtered out at least half of the variants or SNPs with low-quality and inappropriate depth. Next, we calculated *p* value using Ridit analysis at each variant’s position and generated a *p* value plot corresponding to its genomic position (Supplementary Fig. 2a, c, e). Nevertheless, determining a small and precise region was complicated by enormous background noise. Thus, reducing background noise is indispensable. After implementing a noise-reduction algorithm, we narrowed down the interval to 400 kb and successfully localized several intervals harboring causative genes from the ratio plot (Supplementary Fig. 2b, d, f). Among the identified intervals, *Ghd7* and *sd1* (refs. ^17,35^) are exactly located in our mapping regions, and the *GW6a* gene^36^ is located in a position closely adjacent to the mapping interval on chromosome 6. These results demonstrate that the GPS method can rapidly and accurately identify QTLs underlying the target traits. Other identified genetic regions not consistent with any known genes might harbor new QTLs, although further verification is needed.

Moreover, we evaluated the power of GPS to detect regions responsible for heading date, tiller angle and flag leaf angle. The phenotype categories for the F_2_ generations are listed in Supplementary Table 1, and ratio plots are presented in Supplementary Fig. 3. The results for these four agronomic traits all suggested robust applications of our method (Table 1). We then performed GPS to analyze Takagi et al.’s data^29^, comparing with another WGS-BSA method. The results showed consistency with their results, and our mapping region of causal genes was narrowed to 0.4 Mb (Supplementary Fig. 4). Additionally, we explored the whole procedure of our approach in depth, especially the influence on results when changing experimental variables (e.g., pool size, coverage, number of bulks, cases of misclassification and different statistical algorithm). The results of our computer simulation experiment are discussed in the Supplementary Note 1 (Supplementary Fig. 5).

**Table 1:** Identified genomic intervals for four agronomic traits

Trait	Population	Chromosome	Region (Mb) IRGSP 4.0	Known loci
Plant height	1	6	17.2–17.6	
		7	8.8–9.2	*Ghd7* ^a,^ ^17^
		12	22.0–22.4	
	2	6	26.8–27.2	*GW6a* ^b,^ ^36^
		7	6.0–6.4	
		10	17.2–17.6	
	3	1	40.0–40.4	*sd1* ^a,^ ^35^
		9	18.0–18.4	
Heading date	1	5	22.4–22.8	
		7	8.8–9.2	*Ghd7* ^a,^ ^17^
	4	8	3.6–4.0	*Ghd8* ^b,^ ^18^
Tiller angle	2	9	21.2–21.6	*TAC1* ^a,^ ^60^
	3	1	42.4–42.8	
		8	24.8–25.2	*IPA1* ^b,^ ^22^
		9	21.2–21.6	*TAC1* ^a,^ ^60^
Flag leaf angle	3	1	40.8–41.2	*sd1* ^b,^ ^35^
		8	24.8–25.2	*IPA1* ^b,^ ^22^
		9	16.8–17.2	*DEP1* ^a,^ ^61^

^a^The known genes located in identified genomic intervals via GPS

^b^The known genes located closely adjacent to the identified intervals via GPS

### Cloning and functional analysis of the QTL *OsMADS1*^*GW3p6*^

In our previous study, we mapped a genomic region containing the QTL *GW3p6* contributing to the high grain production of the elite hybrid rice variety Guang-Liang-You 676 (GLY-676) from F_2_ individuals^16^. To further fine clone the QTL *GW3p6*, we applied GPS to the F_2_ population derived from the elite hybrid rice variety GLY-676 (heterozygous first filial (F_1_)), which was generated from a cross between the varieties FH (male line) and GZ (female line).

**Fig. 2:**
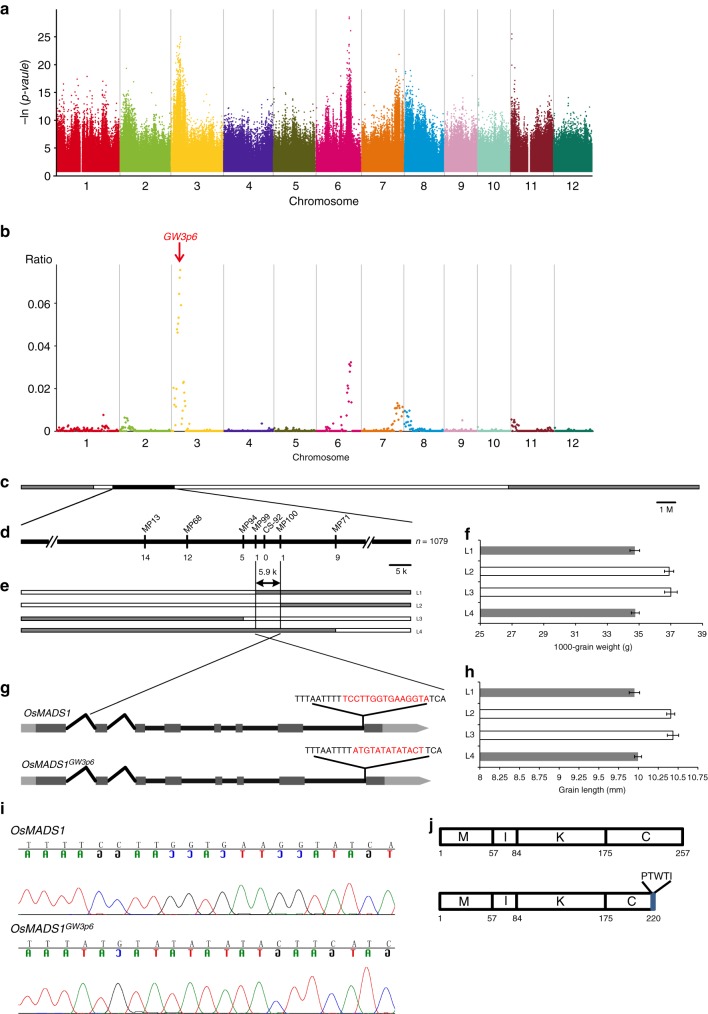
QTL mapping and map-based cloning of *OsMADS1*^*GW3p6*^. **a**, **b** Identification of the genomic region consisting of *OsMADS1*^*GW3p6*^ via GPS approach. **a** The *p* value plot is the result of Ridit analysis for 1000-grain weight before noise-reduction algorithm. The -ln (*p* value) plot (*Y* axis) is plotted against SNP positions (*X* axis) on each of the 12 rice chromosomes. **b** After the strategy of reducing background noise, the results present as ratio plot. *X*-axis value is set at a midpoint at each defined genomic interval and *Y*-axis value corresponds to ratio. **c** The genotype of chromosome 3 of RIL-79. Black, white and gray bars represent the heterozygous genotype, and homozygous genotypes of GZ and FH respectively. **d** In the fine-scale mapping (lower bar) generated from the analysis of 1,079 segregating individuals, the QTL *GW3p6* falls in the heterozygous interval. The numbers below the bar indicate the number of recombinants between *GW3p6* and the molecular markers shown. **e** Genotyping of progeny homozygous for *GW3p6* delimited the locus to a ~5.9 Kb genomic region between markers MP99 and MP100. **f**, **h** 1000-grain weight and grain length of recombinant F_2_ lines of RIL-79 (L1-L4). Data shown as means ± SD, *n* = 48. Source data are provided as a Source Data file. **g** Schematic diagram depicting the structure of *OsMADS1* and *OsMADS1*^*GW3p6*^, red letters stand for the nucleotides of *OsMADS1* non-homozygous segment. **i** The sequencing electrophoresis of non-homologous segment between *OsMADS1* and *OsMADS1*^*GW3p6*^. **j** The schematic illustration of *OsMADS1* functional domains, M represents the MADS domain, I represents the intervening domain, K represents the keratin-like domain, and C represents the C-terminal domain. Source data of Fig. 2f, h are provided in a Source Data file

First, we selected 1000-grain weight (TGW) as the trait for mapping heterotic genes. We ranked three categories (22.33–29.15 g/1000-grain, 29.16–31.09 g/1000-grain and 31.10–37.3 g/1000-grain) to phenotype TGW according to traits and then created simulated pools with their sequencing reads (individual sequencing reads data from European Nucleotide Archive under the accession number PRJEB13735). We implemented Ridit analysis with allelic frequencies from three bulks to calculate *p* values for each SNP (Fig. 2a). As numerous background noises complicated locating the QTL at a fine resolution, a noise reducing strategy followed the statistical test. After conducting all analysis, we located a 400-kb candidate interval contributing to grain weight (Fig. 2b). The mapping accuracy and resolution of GPS can reach almost the same level as that of Composite Interval Mapping. Notably, the GPS mapping results show that the method can be used as a faster and more convenient approach than conventional mapping methods in rice breeding. Considering the compatibility of the different versions of the assembled data, we remapped the TGW genes by GPS method based on Os-Nipponbare-Reference-IRGSP-1.0 (ref. ^37^) and MH63RS2 (ref. ^38^), and the genetic mapping results from the three reference genome assemblies were almost the same (Supplementary Fig. 6).

Furthermore, we screened the recombinant inbred lines (RILs) from the self-pollination F_5_ generation, and RIL79, one RIL in which genomic segment of *GW3p6* was heterozygous but others were homozygous, was selected as a further segregating population (Supplementary Fig. 7). We used 1,079 plants from the F_1_ population of RIL79 to fine-scale map *GW3p6*, and 36 SNP markers were used for genotyping, ultimately narrowed down the interval to a ~5.9-kb region flanked by MP99 and MP100 (Fig. 2c–h). This region contains only the second half of Os03g0215400 (RAP-DB), and further sequencing analysis indicates a 15-bp non-homologous segment at the junction of the seventh intron and eighth exon of Os03g0215400 (Fig. 2g, i), from TCCTTGGTGAAGGTA to ATGTATATATACT. The 3′ terminal bases AG of the seventh intron were altered, and we speculated that this might lead to alternative splicing. The cDNA sequencing data showed that the splice site (AG/GT) slipped to the 32nd nucleotide (AG/GC) of the last exon (Supplementary Fig. 8), directly caused a premature stop codon, and the original mature protein was truncated by 32 amino acid resides (Fig. 2j, Supplementary Fig. 9). We used the Insertion/Deletion (InDel) marker CS-92 to verify the association between grain size and alternative splicing. Totally 200 individuals of each *OsMADS1* genotype were counted, and the heavier grain weight and more slender grain size were in complete agreement with *OsMADS1*^*GW3p6*^ (Supplementary Fig. 10). These results are also consistent with the performance of three *GW3p6* genotypes in the F_2_ generation as previously reported^16^, and heterozygous *OsMADS1*^*GW3p6*^ showed incomplete dominance. These results indicate that this *OsMADS1*^*GW3p6*^ alternative splicing caused by non-homologous segment is responsible for significant grain weight as previously reported^39,40^.

**Fig. 3:**
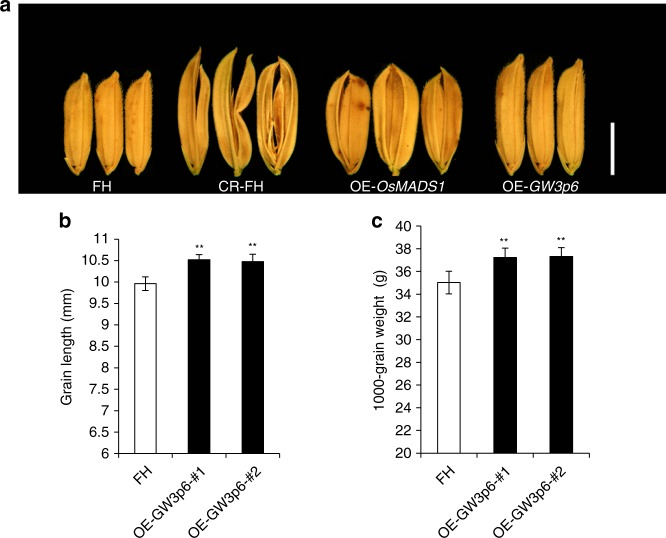
The phenotype of FH transgenic plants. **a** The grain morphology of FH and transgenic plants. FH represents the grain morphology of non-transgenic plant FH. CR-FH represents the grain morphology of transgenic FH plants containing missense mutations in C domain of *OsMADS1*. OE-*OsMADS1* represents the grain morphology of transgenic FH plants containing overexpression *OsMADS1* construct. OE-*GW3p6* represents the grain morphology of transgenic FH plants containing overexpression *OsMADS1*^*GW3p6*^ construct. Scale bar, 5 mm. **b** Grain length in transgenic overexpression plants and non-transgenic plants. OE-*GW3p6*-#1 and OE-*GW3p6*-#2 indicate the two transgenic lines containing overexpression *OsMADS1*^*GW3p6*^ construct. **c** 1000-grain weight in transgenic *OsMADS1*^*GW3p6*^ overexpression plants and non-transgenic plants. Data in (**b**, **c**) are given as means ± SD (*n* = 24) with the indicated significance by using a two-tailed Student’s *t*-test (**p < 0.01). Source data of Fig. 3b, c are provided as a Source Data file

To further examine whether the function of *OsMADS1*^*GW3p6*^ acted as a grain size gene, we transformed FH (male parent) with either rice *Ubiquitin* promoter-driven *OsMADS1* cDNA from FH (p*Ubi*::*OsMADS1*-FH,OE-*OsMADS1*) or *OsMADS1*^*GW3p6*^ cDNA from GZ (p*Ubi*::*OsMADS1*^*GW3p6*^-GZ,OE-*GW3p6*). The grains of the p*Ubi*::*OsMADS1*^*GW3p6*^ transgenic plants were longer and heavier than the non-transgenic control plants (Fig. 3a–c), but constitutive expression of the FH *OsMADS1* cDNA driven by the rice *Ubiquitin* promoter led to abnormalities in lemmas and paleae, as previously described^41,42^ (Fig. 3a). Moreover, we designed the target primers at the last exon of *OsMADS1* using the CRISPR-Cas9 system in FH (CR-FH). Sequencing analysis revealed several insertions and deletions at the last exon in transgenic plants that resulted in loss of function in the C domain of the protein encoded by *OsMADS1*. The transgenic panicles exhibited phenotypic alterations (Fig. 3a), including elongated leafy paleae and lemmas, as well as low fertility seriously as previously reported^40,42^. The result was also similar to the reported phenotype of *leafy hull sterile 1*^41^. Furthermore, the C-terminal function caused changes in glume development. In addition, we transferred the same overexpression construct of *OsMADS1*^*GW3p6*^ and knockout construct by CRISPR/Cas9 system to the *japonica* variety Nipponbare (NPB) with same *OsMADS1* genotype as FH. The overexpressing NPB plants (OE-*GW3p6*-NPB) exhibited longer and heavier grains compared with the non-transgenic plants (Supplementary Fig. 11c, d, e). The transgenic NPB plants carrying missense mutations in the C domain of *OsMADS1* (CR-NPB) exhibited the same phenotype as CR-FH plants (Supplementary Fig. 11a, b, c). The above data imply that *OsMADS1* plays a significant role in the development of rice flower. Typically, the C domain of *OsMADS1* is closely related to the growth of glumes and the development of floral organs, and the function of *OsMADS1*^*GW3p6*^ is to increase grain length and weight.

*OsMADS1* encodes an MIKC^c^-type MADS-box transcription factor^43^; it is also an E-class gene involved in rice floral organ development^44,45^. The MIKC-type MADS-domain proteins consist of four domains: the MADS domain, Intervening domain, Keratin-like domain and C Domain respectively. The C-terminal region has been associated with transcriptional activation^46^, and the non-homologous segment of *OsMADS1*^*GW3p6*^ was located within the C domain. Therefore, we conducted transcriptional activation experiment by yeast one-hybrid system (Supplementary Fig. 12). The results showed that both full-length *OsMADS1* and *OsMADS1*^*GW3p6*^ had no transcriptional activity, which may be associated with a full-length inhibition of its transcriptional activity^46^. In addition, the transcriptional activity of the *OsMADS1* C-terminal region was higher than that of *OsMADS1*^*GW3p6*^. To further verify our experimental results, we applied a fluorescence report system for transcription activation experiments in rice protoplasts. The results were consistent with the data obtained in the yeast system, with *OsMADS1* showing approximately 5 times stronger transcriptional activity than *OsMADS1*^*GW3p6*^ (Supplementary Fig. 13). These data were also consistent with previous reports that alternative splicing attenuates the activation of downstream genes, possibly regulating the transcriptional level of downstream auxin-related genes to change the grain size^39^. The analysis of rice young panicles through RT-qPCR indicated that the expression of *OsMADS1*^*GW3p6*^ was indeed higher than *OsMADS1* (Supplementary Fig. 14). According to our analysis of the structure of *OsMADS1*^*GW3p6*^, a large number of SNPs and InDels are located the upstream of the 5′UTR, and we evaluated whether the observed changes in expression are associated with nucleotides differences in the promoter. To this end, we used the fluorescence report system to verify the promoter, and found no obvious difference between the promoter of *OsMADS1* and *OsMADS1*^*GW3p6*^ (Supplementary Fig. 15). Thus, this change in expression was likely caused by a premature stop codon due to alternative splicing. Taken together, these changes in the C domain may have resulted in the change of grain size.

### Improved grain yield by constructing a NIL containing *GW3p6*

**Fig. 4:**
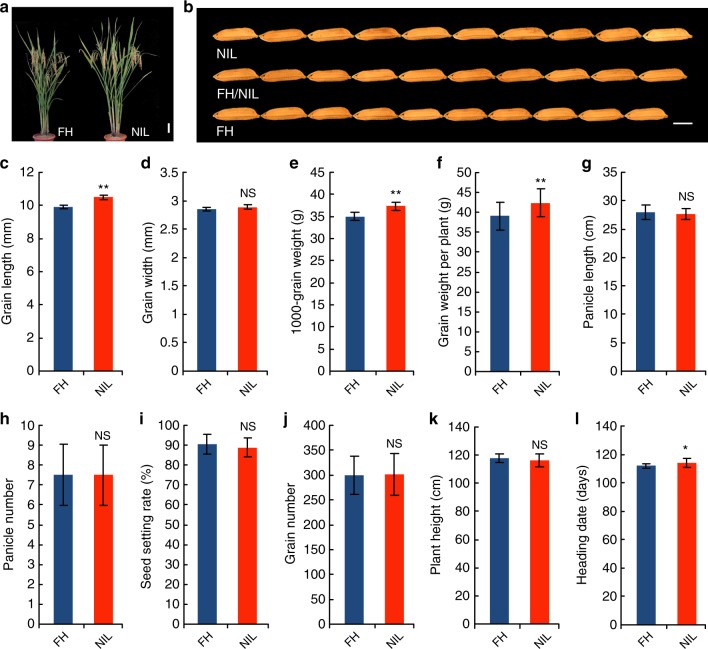
A yield-related traits of FH and NIL-FH: *GW3p6*. **a** Plant phenotype of FH and NIL-FH::*GW3p6*. Scale bar, 10 cm. **b** Grain morphology of FH, NIL-FH:*GW3p6* and FH/ NIL-FH::*GW3p6*. NIL and FH represent the grain morphology of NIL-FH::*GW3p6* and FH, respectively. FH/NIL indicates the grain morphology of plants with heterozygous *OsMADS1* (*OsMADS1/OsAMDS1*^*GW3p6*^) genotype. Scale bar, 5 mm. **c**–**l** A field-based comparison of the FH and NIL-FH::*GW3p6* plants. **c** Grain length, **d** grain width, **e** 1000-grain weight, **f** grain yield per plant, **g** panicle length, **h** panicle number, **i** seed-setting rate, **j** grain number per panicle, **k** plant height, and **l** heading date. Data are shown as means ± SD (*n* = 24) with the indicated significance from a two-tailed Student’s *t* test. For statistical significance, *p < 0.05, **p < 0.01, NS not significant. Source data of Fig. 4c–l are provided as a Source Data file

To further investigate the genetic function of *GW3p6*, the near-isogenic line NIL-FH::*GW3p6* was generated by introgression of *GW3p6* in the FH background. Some RILs with the genetic background of FH accounting for the vast majority were selected as backcrossing materials to generate NILs and were backcrossed twice to FH. With the aid of screening using a large number of molecular markers, and ultimately through sequence-based high-throughput genotyping, we generated a NIL with the FH genetic background and a ~130-kb heterozygous segment. Meanwhile, due to the heterozygous genotype on *GW3p6*, we can observe phenotypic changes in the three *GW3p6* genotypes among the offspring, and the phenotype of incomplete dominance could be observed (Fig. 4b). In general, the phenotypes of NIL-FH::*GW3p6* were similar to that of FH (Fig. 4a), including grain width, panicle number, panicle length, seed-setting rate, grain number per panicle and plant height (Fig. 4d, g–k), though the grain length and 1000-grain weight of NIL-FH::*GW3p6* were ~6–7% higher than those of the FH plants (Fig. 4c, e). In addition, the grain yield per plant of NIL-FH::*GW3p6* was increased by more than 8% (Fig. 4f), while the heading date had a 1~2 days delay compared to that of FH (Fig. 4l). Thus, *GW3p6* is a useful target gene in breeding. By constructing a NIL, we demonstrated that an introgression line harboring a heterosis gene from the maternal parent could achieve better performance than inbred line. It also proved rice heterosis genes’ incomplete dominance played an important role in hybrid rice (Fig. 4b).

### The heterosis effect of *OsMADS1*^*GW3p6*^ in rice breeding

**Fig. 5:**
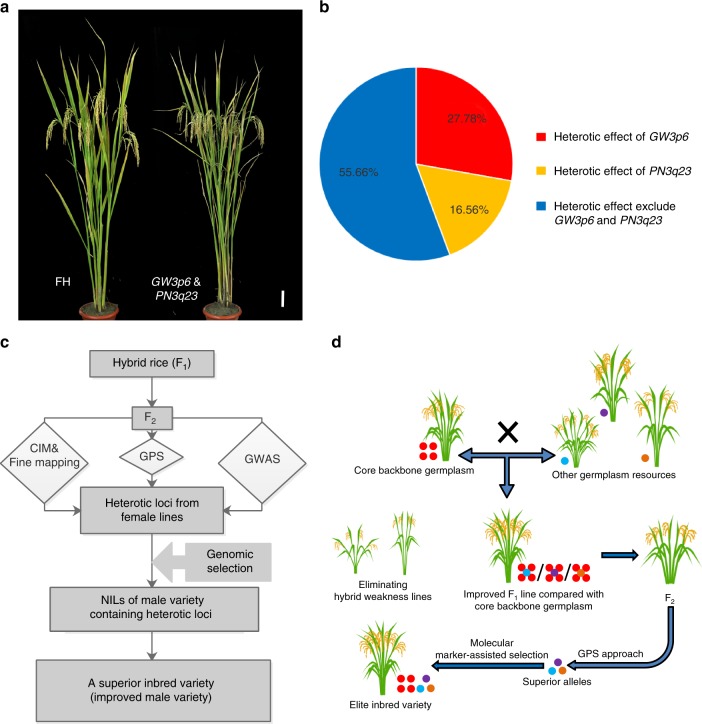
The heterosis effect of *OsMADS1*^*GW3p6*^ in rice breeding. **a** The phenotype of FH plants and plants carrying two heterotic genes *GW3p6*&*PN3q23*. Scale bar, 10 cm. **b** The yield-related contribution of *GW3p6* and *PN3q23*. The proportion of *GW3p6* and *PN3q23* in yield-related heterosis effect are indicated in the pie chart. The color red and orange represent the heterosis contribution rate of *GW3p6* and *PN3q23* respectively. The color blue indicates the heterosis contribution rate of heterotic genes exclude *GW3p6* and *PN3q23*. **c** The flow chart of improvement breeding about hybrid rice in this study. **d** The strategy of rice-improvement breeding in conventional rice and hybrid rice. Colored circles represent superior alleles, for example, the blue, purple and brown circles denote superior alleles associated with tiller number, panicle type and plant architecture. Souce data of Fig. 5b are provided as a Source Data file

As shown above, the NIL carrying the heterotic gene *OsMADS1*^*GW3p6*^ showed significantly increased grain yield. To further explore the potential of *OsMADS1*^*GW3p6*^ in rice breeding, we pyramided another previously reported heterotic QTL *PN3q23* underlying panicle number^16^. The plants harboring two heterotic genes exhibited ~15% increased grain yield compared to the FH plants (Fig. 5a, Table 2), as well as higher yield than NIL-FH::*GW3p6* plants (Table 2). The panicle number of the plants harboring *PN3q23* was also significantly increased compared with that of FH (Supplementary Table 2). We measured the yield per plant for FH, NIL-FH::*GW3p6* and GLY676 respectively. The results exhibited that *GW3p6* explained 27.8% of the heterotic effect (Fig. 5b). The *GW3p6* and *PN3q23*, two major heterotic genes from the female parent, explained over 40% of the heterotic effect (Fig. 5b). These findings implied a few heterotic genes from female parent played important roles in heterosis. We detected the haplotype of *OsMADS1*^*GW3p6*^ in 1328 varieties of hybrid rice (Supplementary Data 1) simultaneously. *OsMADS1*^*GW3p6*^ was rarely detected among the three-line type hybrids, in which the proportion was ~1.6%. Approximately 11.5% of the two-line type hybrid varieties were found to carry the *OsMADS1*^*GW3p6*^ allele. These data indicate there is a large breeding potential for application of the superior allele of *OsMADS1*^*GW3p6*^ in future hybrid rice breeding.

**Table 2:** Grain yield per plant of different plants harboring heterosis genes

(I) Genotype	No. of plants	Grain yield per plant (g)^a,b^	(J) Genotype	Mean differences (I-J)	Standard error	Significance (*p* value)^c^
FH	70	42.644 ± 2.796	GLY-676	−14.234	1.222	4.319 × 10^−14^
			NIL-FH::*GW3p6*	−3.971	0.726	7.267 × 10^−7^
			*GW3p6* + *PN3q23*	−6.323	0.726	7.894 × 10^−14^
NIL-FH::*GW3p6*	70	46.615 ± 2.853	GLY-676	−10.263	1.222	1.082 × 10^−13^
			FH	3.971	0.726	7.267 × 10^−7^
			*GW3p6* + *PN3q23*	−2.352	0.726	7.479 × 10^−3^
*GW3p6* + *PN3q23*	70	48.968 ± 6.330	GLY-676	−7.911	1.222	3.662 × 10^−14^
			FH	6.323	0.726	7.894 × 10^−14^
			NIL-FH::*GW3p6*	2.352	0.726	7.479 × 10^−3^
GLY-676	15	56.878 ± 3.888	FH	14.234	1.222	4.319 × 10^−14^
			NIL-FH::*GW3p6*	10.263	1.222	1.082 × 10^−13^
			*GW3p6* + *PN3q23*	7.911	1.222	3.662 × 10^−14^

^a^All data is given as means ± SD

^b^Data for the grain yield per plant are based on a field experiment using a randomized complete block design

^c^*p* value produced by the Tukey’s HSD

^I,J^Grain yield per plant of plants with different genotype

In conclusion, the heterotic gene *GW3p6* can improve the grain yield significantly in hybrid rice. By dissecting the heterotic gene *OsMADS1*^*GW3p6*^, we summarized a rice breeding strategy in hybrid rice and inbred rice (Fig. 5c, d). The F_1_ progeny derived from different crosses exhibited heterosis in certain agronomic traits, and we could locate superior genes in their F_2_ generations through the rapid and convenient GPS method, finally achieving fast and precise breeding through the marker-assisted selection approach.

## Discussion

Traditional QTL mapping methods depending on the genetic linkage of QTLs to visible makers are laborious and time-consuming. The advent of NGS technologies and BSA has offered new opportunities for rapid identification of QTLs. Several methods have been established to accelerate the works in genetic mapping. We developed GPS, an improved approach combining high-throughput sequencing with modified BSA, for QTL mapping in crop breeding. Our method has several advantages over several previous methods. First, instead of using mutant lines such as Mutmap^26^ and Next-generation mapping^28^, we choose parental lines comprising a large quantity of useful alleles directly. Second, our approach only requires F_2_ generation, reducing a great amount time needed for constructing genetic population. Third, GPS has a high resolution of ~400-kb, whereas the resolution of QTL-seq^29^ is ~2 Mb. In this work, we successfully identified the QTLs underlying five target traits (heading date, plant height, tiller angle, flag leaf angle and grain weight) in rice, demonstrating that GPS has a robust and extensive applicability for QTL mapping. In addition, the cost-effectiveness of the entire GPS process is relatively high compared with other methods. With the decline in high-throughput sequencing, applying GPS to identify QTLs would be economical. GPS can map QTLs underlying multiple traits simultaneously in one rice population. For example, a leaf sampled once can be used multiple times. Moreover, GPS does not require genotyping of all individuals, saving both time and laborious effort. Overall, this method significantly enhances the efficiency and cost-effectiveness of mapping candidate genes, enabling rapid identification of heterotic genes for rice breeding. Breeders and researchers would find good trade-offs in cost-effectiveness due to the relatively broad requirements and high efficiency of GPS. Thus, the highly efficient GPS will dramatically accelerate crop improvement in a cost-effective manner.

GPS shows a relatively wide range of applications in fine-mapping and breeding, but some limitations are also existed in GPS approach. First, QTL by environment interaction (QEI) is widely present in crops and other species. GPS with Ridit analysis currently had low power in QEI. Second, the GPS pipeline may not work very well with the overdominant loci. To identify QTLs through the software GPS, the SNPs related to the agronomic traits need to have a great distinction of reference reads to alternative reads. However, for the overdominant loci, there may be a great distinction of heterozygous genotypes to homozygous genotypes, but less great distinctions of reference reads to alternative reads. These limitations of GPS should be accounted for and assessed in plant breeding and fine mapping.

In our simulation experiment, we consider five experimental variables that influence the results of accurate detection. In the first place, we took the possibility that unlinked SNPs were calculated as related one into consideration. The simulation indicated that the number of individuals in each bulk had an impact on it. The more individuals we selected in each group, the less likely errors of false positive occurred. Furthermore, the number of pools is another important variable in our method. Meanwhile, more bulks meant being less influenced by incorrect categorization, thus we needed a balance here. In the simulation of the effects of misclassification, we took the 20% wrong classification of individuals in a certain bulk as an example, and this would be the main element leading to the inaccuracy of results. Three factors, depth, number of selected individuals and number of bulks, together influenced the capability of our method to detect the QTL within the context of misclassification. With the increasing value of these experimental variables (depth, pool size, number of bulks), the power to uncover the QTLs becomes larger. Finally, we also assessed the results of different statistical algorithms and determined the optimal one. It turned out that the Ridit analysis had the best performance in identifying QTL among them.

As shown above, the near isogenic line NIL-FH::*GW3p6* displayed a large increase in yield compared with FH, but it was still slightly less productive than the F_1_ plants. The reason for this phenomenon is mainly there are multiple heterosis genes from maternal parents. With the benefits of accurate QTL mapping, GWAS and an improved GPS tool will be able to rapidly identify heterotic genes for crop breeding. Further studies are needed to identify and pyramiding more heterotic genes with regard to various yield-related traits in important backbone parent, such as the tiller number related gene *PN3q23*.

The incomplete dominance effect of *OsGW3p6* may be due to an allelic dosage effect as in maize^47^. Uncovering more alleles and fine-tuning the dosage of *OsMADS1*^*GW3p6*^ may produce an optimal yield as previously study in tomato^48,49^.

To explore the potential of rice breeding, we also sequenced the genome DNAs of hybrid rice parents (Supplementary Data 2). The results showed that in two-line hybrid rice, there are three male-sterile lines carried *OsMADS1*^*GW3p6*^, i.e., M6303S, Xuan69S and Guangzhan63S. In three-line hybrid rice, the proportion of *OsMADS1*^*GW3p6*^ is small. Moreover, *OsMADS1*^*GW3p6*^ was present in the restorer line, such as Nanhui511, a big spike-type *indica* restorer line with broad adaptability. A phylogenetic tree of 1,473 *indica-indica* hybrid rice varieties showed that over half of those containing *OsMADS1*^*GW3p6*^ were clustered with the hybrid rice GLY-676 (Supplementary Fig. 16), which may be associated with a similar genotype grouped in the same clade. Using rice pan-genome data^50^, we found that *OsMADS1*^*GW3p6*^ mainly existed in the tropical *japonica* varieties among 66 rice accessions (Supplemental Fig. 17). These data indicate that *OsMADS1*^*GW3p6*^ has not been exploited in large-scale rice breeding and that the *OsMADS1*^*GW3p6*^ allele has great potential in rice breeding, particularly in hybrid rice. Either as a male-sterile line or restorer line, O*sMADS1*^*GW3p6*^ will have a huge impact on rice breeding.

In order to meet the growing demand, rice hybrid breeding requires additional superior germplasm resources to enhance yield potential, and future molecular breeding will need to overcome inter-subspecific hybrid sterility to broaden genetic diversity and enhance heterosis^51^, uncovering more heterotic genes for use in molecular breeding. We believe the GPS method and rice-improvement breeding strategy reported here will promote hybrid rice breeding.

## Methods

### Plant materials and trait measurement

In the GPS study, four sets of elite hybrid rice were used as mapping populations. The F_2_ generations were obtained by self-pollinating F_1_ hybrid rice. The F_2_ populations with a large range of phenotypic variations were used for identifying QTLs for four agronomic traits (plant height, heading date, flag leaf angle, tiller angle). The four sets of F_2_ populations were planted in a rice paddy located in Hainan Province during the standard growing season, and phenotypic investigations in the field were conducted in the appropriate growth stage in spring of 2017.

In the fine-mapping study, Fuhui676 (FH), an elite *indica* restorer line, and the Photo-Thermo Sensitive Genic Male Sterile Line Guangzhan63-4S (GZ) were used as the recurrent and donor parent respectively, to develop the backcross population and NILs. The RILs that the genetic background of FH accounts for the vast majority were selected as backcross materials to generate NILs. In particular, RIL-81, an F_6_ RIL derived from the cross between FH and GZ, carries a heterozygous segment harboring *OsMADS1*^*GW3p6*^. RIL-81 was backcrossed twice to FH. By screening a large number of molecular markers, and ultimately through sequence-based high-throughput genotyping, we generated the FH genetic background NIL-FH::*GW3p6* containing a ~130-kb heterozygous segment. Similarly, allelic combinations of *OsMADS1*^*GW3p6*^ and *PN3q23* were selected from the cross NIL-FH::*GW3p6* and Chromosomal Segment Substitution Line containing the *PN3q23* segment. All of the plant materials were cultivated in the experimental field in Shanghai in summer or Sanya in winter. Field-grown NIL plants and gene pyramiding plants were grown in a rice paddy at an interplant spacing of 30 × 16 cm during the standard growing season at experimental fields located in Sanya (Hainan Province, China).

Grain length and width were measured using an automatic digital grain size scanner, and fully filled grains were used for measuring 1000-grain weight. Ten plants in the middle of each row were harvested individually and used to investigate yield-related traits, such as panicle number and grain weight per plant. The phenotype data for plant height, panicle length, grain number per panicle and seed-setting rate were obtained using the main culm of each plant. The phenotype of NIL-FH::*GW3p6* plants was investigated in spring of 2018 in Hainan, China. The phenotype of plants containing *GW3p6* and *PN3q23* was investigated in autumn of 2018 in Shanghai, China.

### Bulked-segregant analysis

To validate our method, we chose four complex traits to conduct proof-of-principle experiments. As illustrated in Supplementary Table 1, four agronomic traits were observed in the four sets of F_2_ populations. For plant height, according to phenotypic values, we classified F_2_ populations into several bulks from high to low plant height. The same strategy was carried out with the other three traits using their F_2_ progeny. The number of pools depends on the grade of the phenotypic difference and the number of F_2_ individuals. The phenotypic values of F_2_ individuals were arranged in ascending or descending order. The F_2_ individuals corresponding to the phenotypic grade were marked and assigned to different pools.

In the process of identifying heterotic gene *GW3p6*, we divided the individuals into three ordinal pools according to 1000-grain weight (22.33–29.15 g/1000-grain, 29.16–31.09 g/1000-grain, 31.10–37.3 g/1000-grain), and pool size was 351 (33.56% of the total F_2_ individuals), 348 (33.27%) and 347 (33.17%), respectively.

### Whole-genome sequencing of bulked DNA

For each pool, genomic DNAs were extracted from the fresh leaf tissue of F_2_ individuals using DNeasy Plant Mini Kit (Qiagen). The equal masses of fresh leaves (~0.05 g) were used for DNA pool sequencing. Equal masses of fresh leaves from F_2_ individuals in each pool were mixed in a mortar, and then the genomic DNA of them was extracted for further sequencing. After that, the genomic DNA was fragmented by ultrasonic treatment. A sequencing library was constructed with an insert size of 400–500 bp for a single index according to the protocol of KAPA Hyper Prep Kit (Illumina^®^ platforms). The indexed DNA samples of each pool were then purified using a silica membrane column, followed by size-selection agarose gel electrophoresis (Bluepinpin). The DNA library of each pool was loaded into one lane using the Illumina Hiseq2500 system. In total, 2889 individuals in 37 lanes were sequenced, generating 100-bp paired-end reads. Alignment against the reference genome sequence (IRGSP releases build 4.0 pseudomolecules of rice; Os-Nipponbare-Reference-IRGSP-1.0; MH 63RS2) was performed using BWA software^33^, followed by SNP-calling using GATK^34^ Best Practices (https://software.broadinstitute.org/gatk/best-practices/).

### Filtering process for the sequencing data

Before applying statistical tests to sequencing data, filtering process should be accomplished to guarantee accuracy. The four aspects of filtering criteria are as follow: (1) filtering out of low-quality variants; (2) selecting variants with appropriate depth; (3) screening out variants for which both parental lines present homogeneous and different genotypes; (4) filtering out of the SNPs for which sequence reads from all pools only showed non-reference bases. Statistical analysis was then applied to reveal causative variants.

### Applying statistical test

After filtering process, we coped with sequencing data by statistical tests. Comparing with three nonparametric methods here, Ridit analysis^32^, Kruskal-Wallis Test^52^, and Chi-square test^53^, we chose the optimize one-Ridit analysis. Calculating *p* value of each variant, we pictured *p* value plot by using –ln (*p* value) and chromosomal position as *y*-value and *x*-value, respectively.

### Reducing background noise scheme

Even though we obtained the *p* value of each variant, this was not enough to pinpoint the QTL intervals. We implemented a reducing background noise scheme. Computing the ratio of number of statistically significant variants to total number of variants in a defined interval (~400-kb), while skipping interval where the number of total SNPs is less than 10. We identified the candidate genetic intervals responsible for phenotype via ratio plot. The location of peak represents a cluster of highly linked variants to a given trait in this 400-kb region.

### Genotyping and fine mapping of *OsMADS1*^*GW3p6*^

Plant materials’ genotyping were mainly based on Applied Biosystems 3730 XL fully automatic DNA sequencer to identify SNPs, and high-throughput genotyping by whole-genome resequencing was used to confirm the background genotype^54^. The high resolution melting curve analysis by Roche LightCycler 480 and other genotyping methods (SNPs genotyping by Sanger Sequencing and InDels genotyping by gel electrophoresis) were used to genotype for constructing NIL-FH::*GW3p6*.

Fine-scale mapping of *OsMADS1*^*GW3p6*^ was based on 1,079 F_1_ individuals of RIL-79. 36 SNP markers were used to screen the recombinants. Finally, the *OsMADS1*^*GW3p6*^ locus was narrowed down to a 5.9-kb region between markers MP99 and MP100. The phenotype of grain shape and grain weight in selected recombinants was confirmed using the self-progeny test. The genomic DNA of candidate *OsMADS1* genes from GZ, FH and other landrace varieties were sequenced and analyzed by Sanger Sequencing System. A list of the markers used for fine-scale mapping and NIL construction were given in Supplementary Table 3 and Supplementary Table 4.

### Plasmid construction and plant transformation

To generate the overexpression vector, full-length coding sequence without the stop codon of *OsMADS1* and *OsMADS1*^*GW3p6*^ were cloned into the vector pNCGR-OX fused with His-tag. And to construct sgCRISPR-Cas9 vector, one CRISPR/Cas-mediated target in C-Domain was cloned into CRISPR/Cas9 vector^55^. All the constructs were transformed to *japonica* cv. Nipponbare and *indica* cv. FH by Agrobacterium-mediated transformation. The relevant PCR primer sequences are given in Supplementary Table 5.

### RNA extraction and quantitative RT-PCR analysis

The total RNA was extracted from rice young panicles using TRIzol regent (Invitrogen), and 0.5 micrograms of total RNA was used to synthesize first-strand cDNA by the ReverTra Ace^®^ qPCR RT Master Mix with gRNA Remover (Code NO.FSQ-301, TOYOBO). Quantitative real-time PCR was performed from cDNA using THUNDERBIRD^®^ SYBR^®^ qPCR Mix (QPS-201, TOYOBO) according to the manufacturer’s instructions on Applied Biosystems Q5. The rice *ubiquitin 5* gene was used as an internal control, and each qRT-PCR assay was performed at least three times in biological and technological replicates. The relevant PCR primer sequences are given in Supplementary Table 5.

### Transcriptional activation assay in yeast

The full-length and different truncations or deletions coding sequence of *OsMADS1* and *OsMADS1*^*GW3p6*^ were introduced into the pGBKT7 (Clontech) vector to fuse the GAL4 DNA-binding domain. The empty pGBKT7 vector was as negative control. Then the vectors were transformed into yeast strain AH109 and the clones were diluted to an absorbance of 1.0 (1/10, 1/100, 1/1000) at OD_600_. 15 μl of liquid culture was plated on the control medium and SD/-Leu-His-Ade-+ X-α-Gal medium for 3d at 30 °C. The transcription activation assays were conducted according to the Matchmaker GAL4 Two-Hybrid System 3 (Clontech) user manual. The relevant PCR primer sequences are given in Supplementary Table 5.

### Dual luciferase transcriptional activation assay

The C-Domain of *OsMADS1* and *OsMADS1*^*GW3p6*^ were introduced into pGE vector to construct the effector plasmid. The pGE vector was made from pGBKT7 and pRI101-AN (Takara), the GAL4-BD domain from pGBKT7 was inserted into the pRI101-AN driven by 35S promoter. The pGreen-DBmini vector containing upstream activation sequence was design as the reporter plasmid, and then effector plasmids and reporter plasmids were transformed into rice protoplasts. The empty pGE vector was used as a negative control. Each expression assay was performed at least three times in biological and technological replicates^56^.

### Transient expression assays of promoter activity

The prompter fragments of *OsMADS1* (~3.6 kb upstream sequence of the *OsMADS1*) and *OsMADS1*^*GW3p6*^ (~3.9 kb promoter fragment of the *OsMADS1*^*GW3p6*^) were amplified from FH and GZ respectively, and were inserted into pGreen II 0800-LUC vector containing the firefly luciferase gene and the *Renilla* gene^57^. The rice protoplasts were isolated from rice culm of 7~12 days after seeding. Each of the *OsMADS1* promoter-LUC vector was used for transient transformation into rice protoplasts. And the empty pGreen II 0800-LUC vector transferred into rice protoplasts was as negative control. For rice protoplasts transformation, at least four independent transformations were performed for each sample^58^. The relative activity of LUC to REN luciferase was measured by luminometer.

### Calculation of heterotic contribution rate

In total, 15 plants of GLY-676, and 70 plants of FH, NIL-FH::*GW3p6* and NIL-FH::*GW3p6*&*PN3q23* were used to calculate the heterosis effect contribution rate. Heterosis contribution rate was obtained as follows:

1 $${\mathrm{Heterosis}}\,{\mathrm{contribution}}\,{\mathrm{rate}} = \frac{{Y_{\mathrm{NIL}} - Y_{\mathrm{FH}}}}{{Y_{\mathrm{GLY676}} - Y_{\mathrm{FH}}}}$$

Y_GLY676_ represents the average yield per plant of GLY-676 (F_1_), Y_FH_ represents the average yield per plant of FH, Y_NIL_ represents the average yield per plant of NIL-FH::*GW3p6*.

### Phylogenetic analysis

The 1439 *indica-indica* phylogenetic trees were constructed by the published data^15^. The haplotypes of *OsMADS1* were classified into two categories: *OsMADS1* and *OsMADS1*^*GW3p6*^, the iTOL (version 4.2.4)^59^ was used to display and annotate the phylogenic tree, the red annotation indicated the haplotype of *OsMADS1*^*GW3p6*^.

Similarly, the neighbor-joining tree of the 66 rice accessions constructed by PHYLIP and the package MEGA5 was as known data. According to the haplotype of *OsMADS1*, the haplotype of *OsMADS1*^*GW3p6*^ was marked as an asterisk by iTOL in NJ-tree of 66 rice accessions.

### Reporting summary

Further information on research design is available in the Nature Research Reporting Summary linked to this article.

## Supplementary information

Supplementary Information

Peer Review

Reporting Summary

Description of Additional Supplementary Files

Supplementary Data 1

Supplementary Data 2

Source Data

## Data Availability

Data supporting the findings of this work are available within the paper and its Supplementary Information files. A reporting summary for this Article is available as a Supplementary Information file. All other relevant data are available from the corresponding author upon request. All sequencing data that support the findings of this study have been deposited in European Nucleotide Archive (ENA) with the accession code PRJEB30329 [https://www.ebi.ac.uk/ena/data/view/PRJEB30329]. The source data underlying Figs. 2f, h, 3b, c, 4c–l, and 5b, as well as Supplementary Figs. 10a, b, 11d, e, 13, 14, and 15 are provided as Source Data file.

## Code availability

All software codes of the GPS approach are available in GitHub [https://github.com/sctang1991/GPS-pipeline].
