# Supplementary material for: Dissecting a heterotic gene through GradedPool-Seq mapping informs a rice-improvement strategy
Source: Nat Commun. 2019 Jul 5;10:2982. doi: 10.1038/s41467-019-11017-y (PMC6611799; doi:10.1038/s41467-019-11017-y)
Supplement: Supplementary file 1 — Supplementary Information [file 41467_2019_11017_MOESM1_ESM.pdf]

Dissecting a heterotic gene through GradedPool-Seq mapping informs a rice-improvement strategy

Wang *et al.*

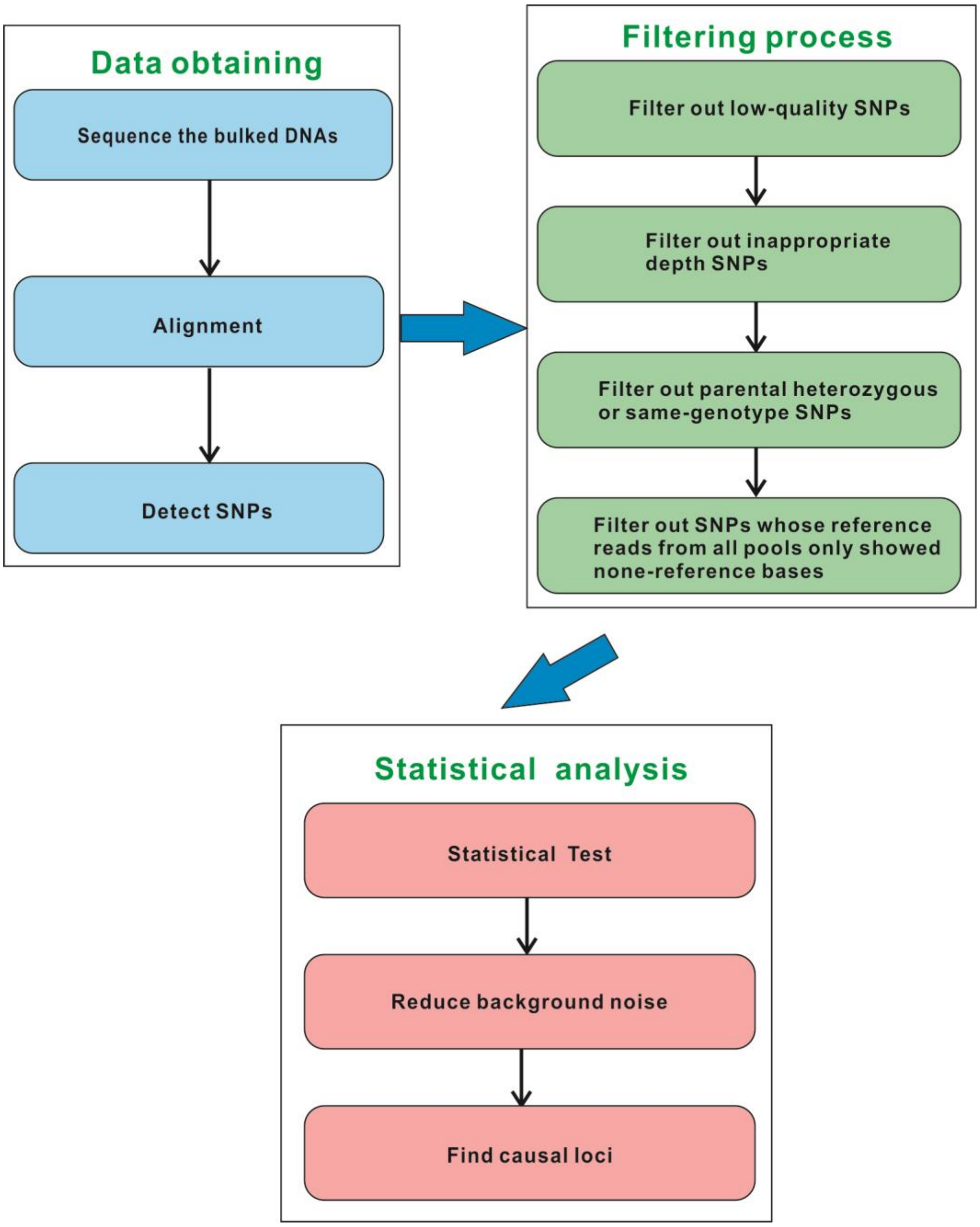

Supplementary Figure 1. The flow chart for data analysis in GPS approach. Three sections, mining genome-wide polymorphism, setting filter criteria and statistical analysis, form the whole procedure of data analysis. The main analysis steps are shown in three boxes.

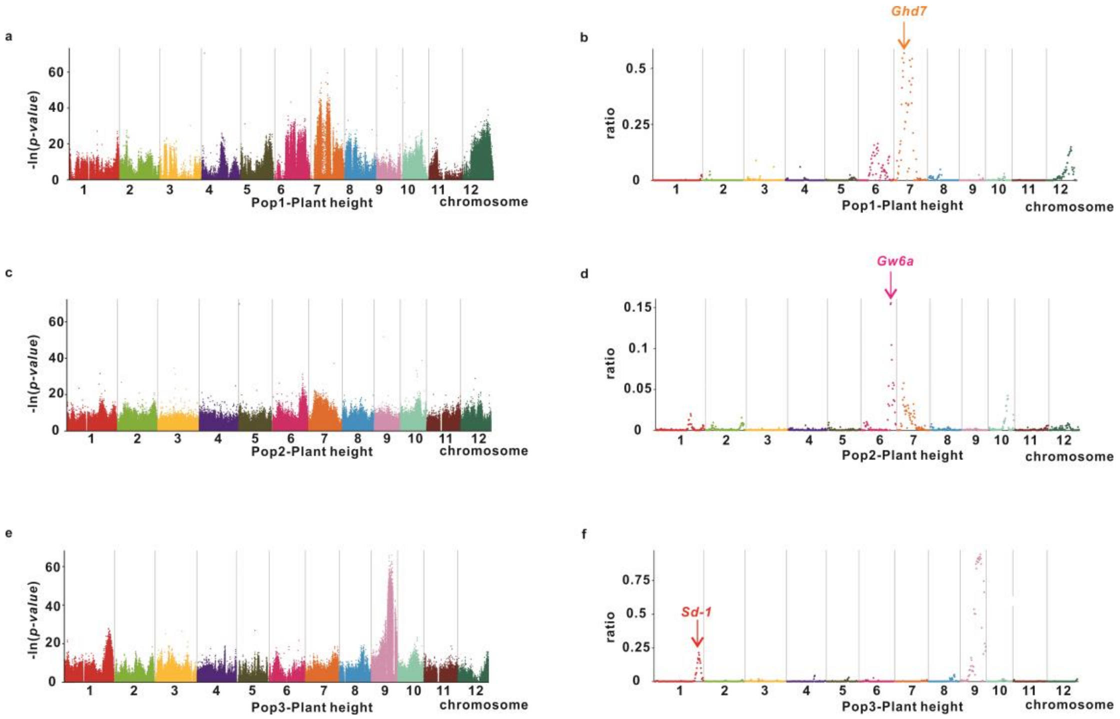

Supplementary Figure 2. *P* value plots and ratio plots for plant height. (**a, c, e**) The results of GPS for plant height in 3 populations before noise-reduction algorithm. The *p* value plots are obtained by Ridit analysis, and the -ln(*p-value*) plots (y-axes) are plotted against SNP positions (x-axes) on each of 12 rice chromosomes. (**b, d, f**) After the noise-reduction algorithm, the results present as ratio plot. X-axes values are set at a midpoint at each defined genomic interval and Y-axes values correspond to ratio. Arrows indicate the position of peak points, and the known genes located in or closed to the peak intervals are labelled upon the arrow.

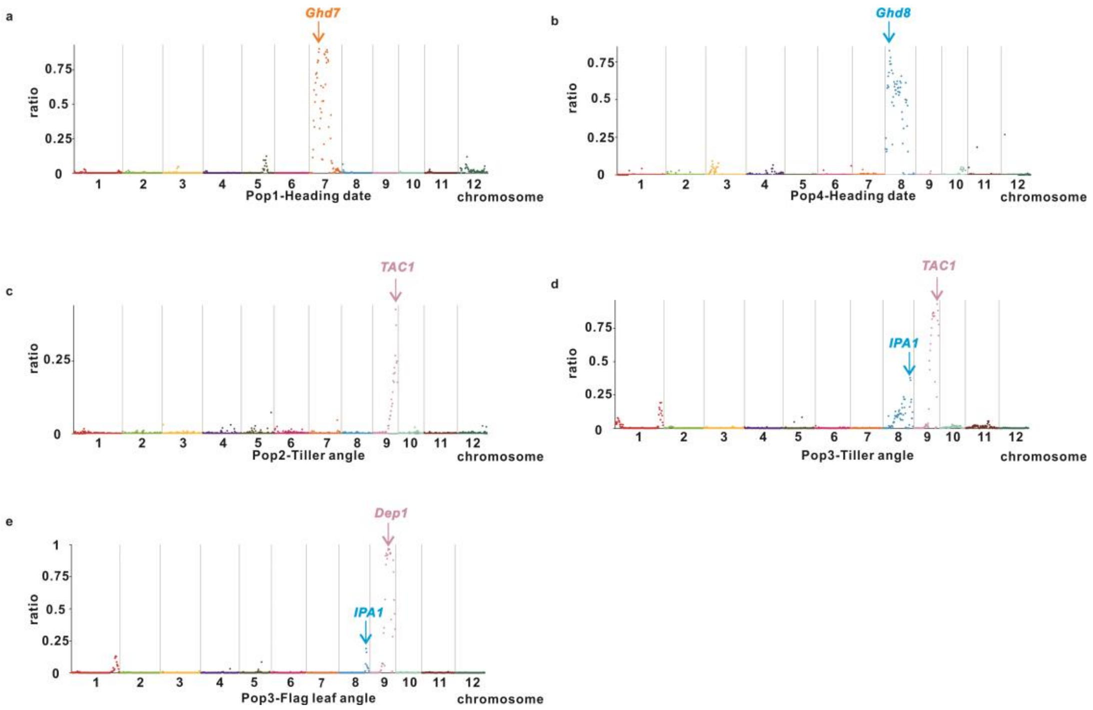

Supplementary Figure 3. Ratio plots of heading date, tiller angle and flag leaf angle. (**a, b**) The results for identifying heading date genes in two populations. (**c, d**) The results for identifying tiller angle genes in two populations. (**e**) The results for identifying flag leaf angle genes in Population 3. X-axes values are set at a midpoint at each defined genomic interval and Y-axes values correspond to ratio. Arrows indicate the position of peak points, and the known genes located in or closed to the peak intervals are labelled upon the arrow.

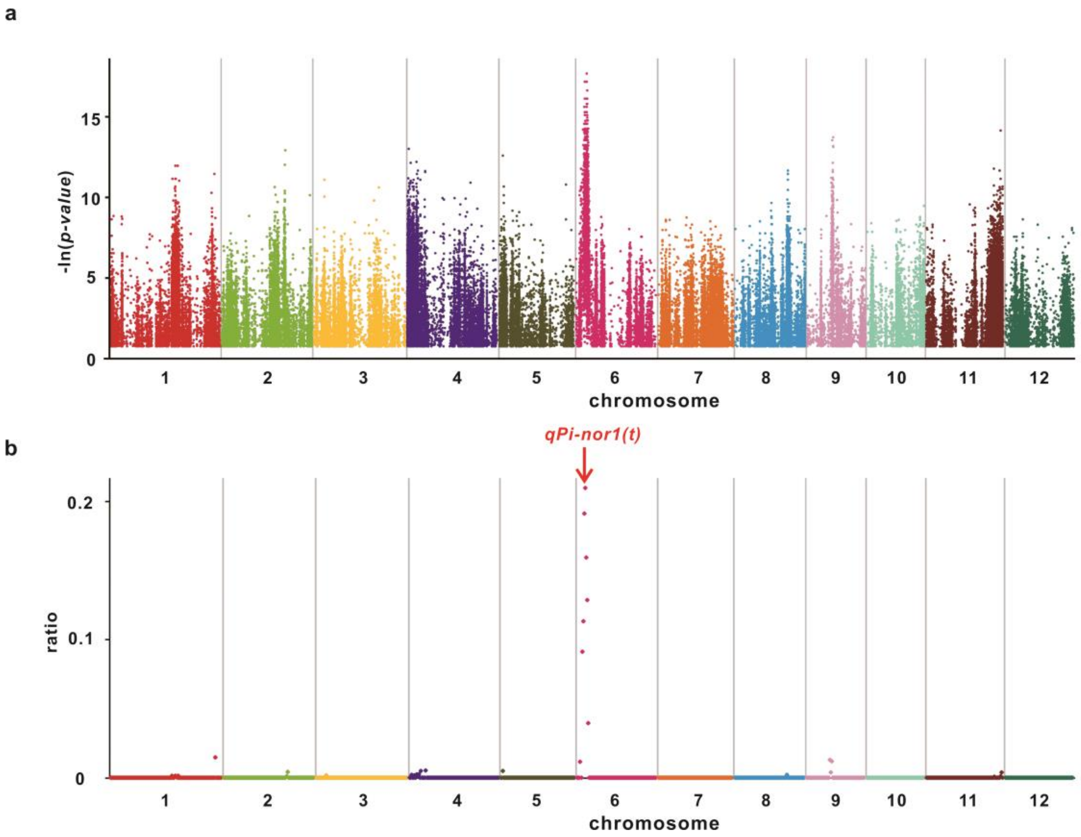

Supplementary Figure 4. *P* value plot and ratio plot of partial resistance to rice blast. GPS was carried out to identify partial resistance genes to rice blast using recombinant inbred lines developed by the cross between Nortai and cultivar Hitomebore. (**a**) The *p* value plot is the results of Ridit analysis for rice blast resistance. The -ln (*p-value*) plot (y-axes) are plotted against SNP positions (x-axes) on each of the 12 rice chromosomes. (**b**) After accomplishing the noise-reduction algorithm, the results present as ratio plot. X-axes values are set at a midpoint at each defined genomic interval and Y-axes values correspond to ratio. Red arrow indicates the peak of genomic interval.

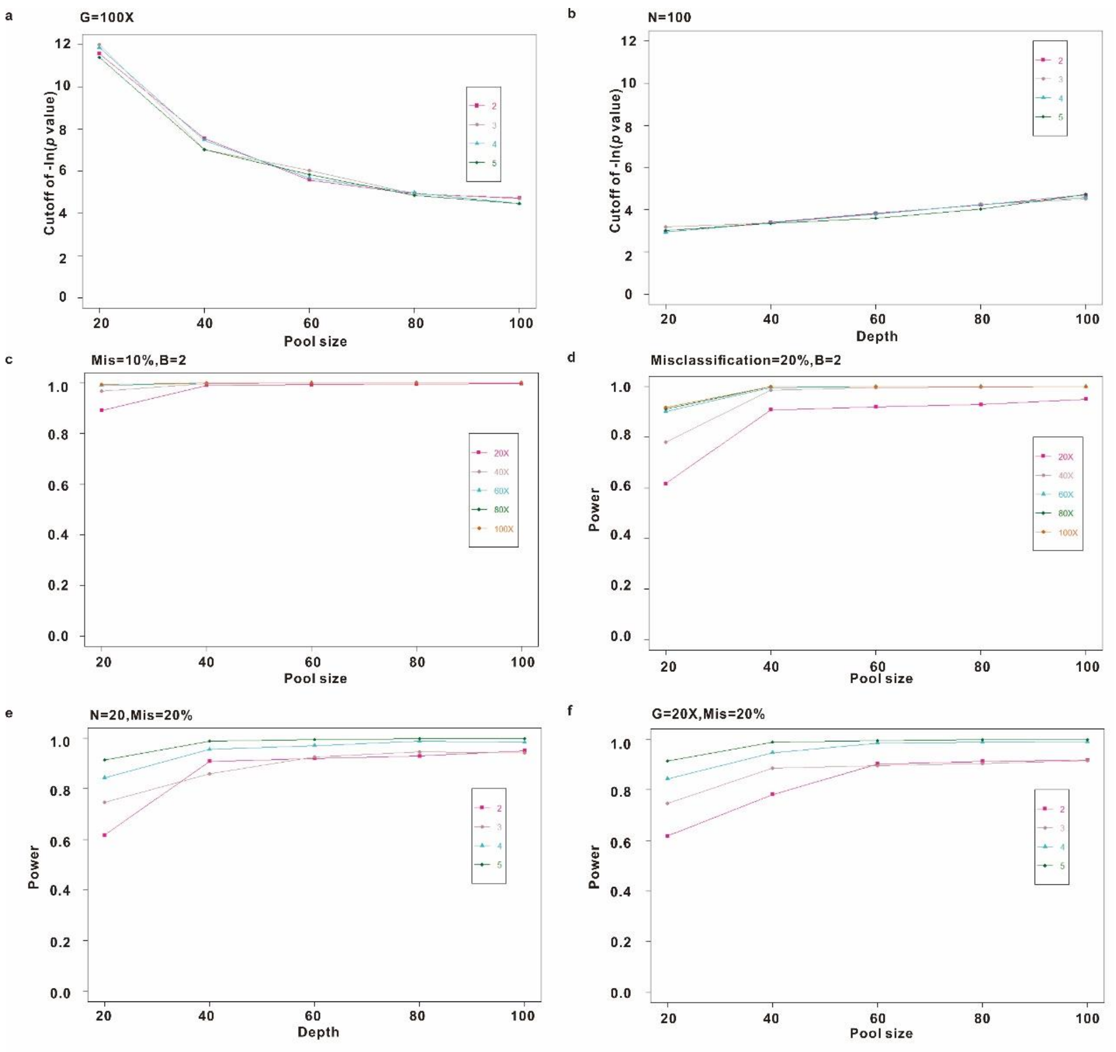

Supplementary Figure 5. The simulation of the GPS experiment.
(**a**) Exploring the influences of pool size (N) and numbers of bulk (B) in the circumstance of false positive when depth G=100×. (**b**) Exploring the influences of G and B in the circumstance of false positive when N =100. (**c**) The capability of detecting QTLs when misclassification of a certain bulk equaled 10%. X-axis and Y-axis represent N and power. (**d**) The capability of detecting QTLs when misclassification of a certain bulk equaled 20%. X-axis and Y-axis represent N and power. (**e**) Changing depth from 20 to 100× to detect the power to identify QTLs when N and Mis were fixed. (**f**) Changing N from 20 to 100 to detect the power to identify QTLs when G and Mis were fixed. The value of the QTL effect is set to 75% in the simulation of GPS.

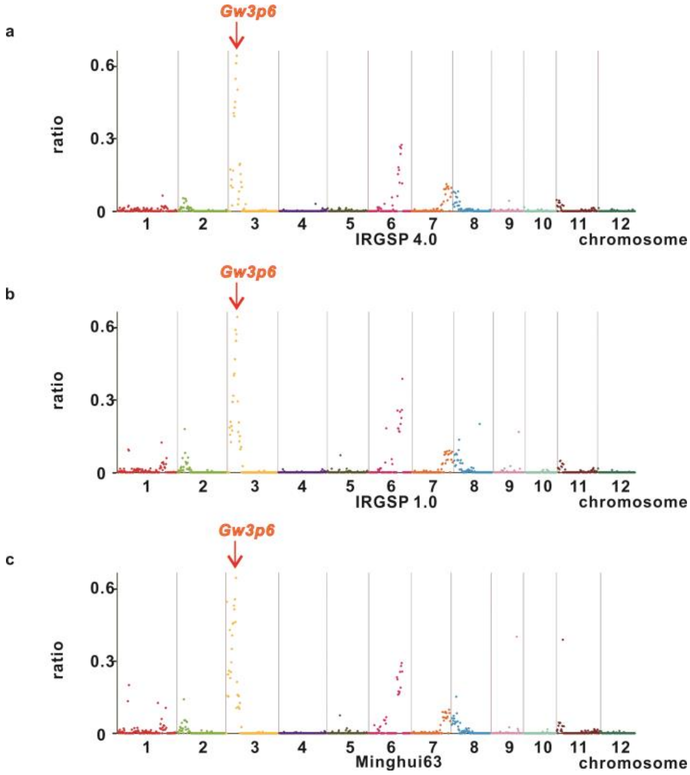

Supplementary Figure 6. The ratio plots of *GW3p6* among different rice genomes. (**a**) The ratio plot results of 1000-grain weight in GLY-676 population based on the IRGSP build 4.0 pseudomolecules of rice. (**b**) The ratio plots of 1000-grain weight based on the IRGSP releases build 1.0 pseudomolecules of rice and (**c**) the genome of Minghui63. X-axes values are set at a midpoint at each defined genomic interval and Y-axes values correspond to ratio. Red arrow indicates the peak of genomic interval.

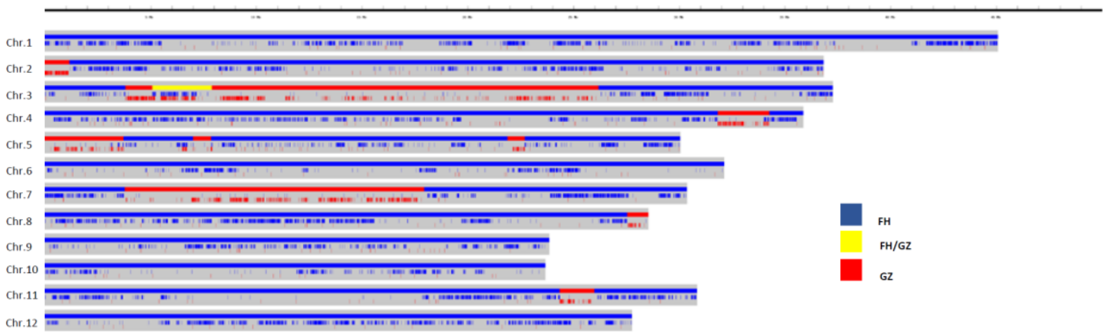

Supplementary Figure 7. The genotype of RIL-79. The recombination map of a single F_6 individual from the cross between FH and GZ by whole-genome sequencing. Three genotypes are showed by blue, red and yellow. Blue represents the homogeneous genotype of FH, red represents the homogeneous genotype of GZ, and the yellow represents the heterozygous genotype of FH/GZ. The genomic DNA of the rice individual is sequenced in the HiSeq 2500 system, and SEG-Map computational algorithm is used to genotype. Detected SNP genotypes are indicated along chromosomes according their specific physical locations. Genotyping calling, recombination breakpoint determination and map construction use the sliding window approach.

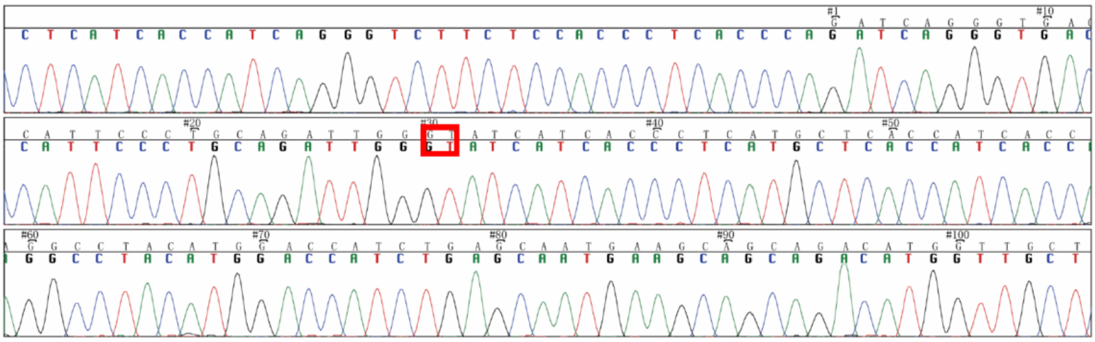

***OsMADS1**^{GW3p6}*

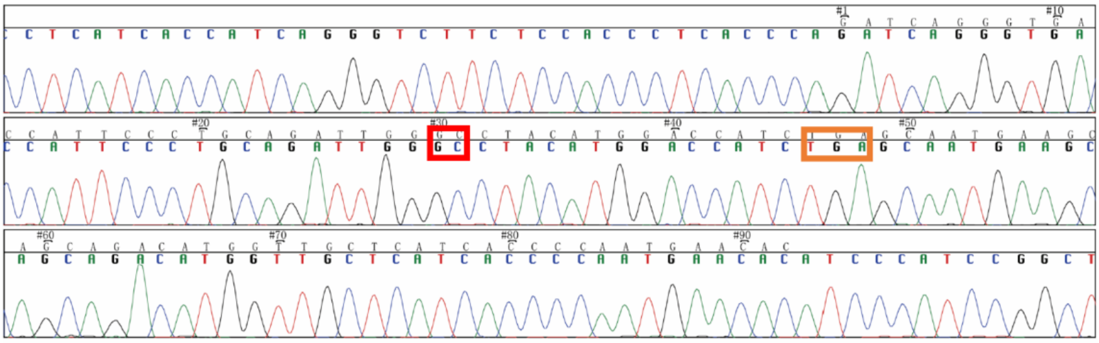

Supplementary Figure 8. The sequencing electrophoresis of cDNA of FH and GZ. The sequencing electrophoresis of cDNA in the position of alternative splicing. The sequencing data were generated by Sanger sequencing approach, and the software sequencher 4.5 was used for assembling the sequencing results. The red box represents the recognition site of splicing (AG/GT and AG/GC), the brown box represents the nucleotides corresponding to termination codon (TGA).

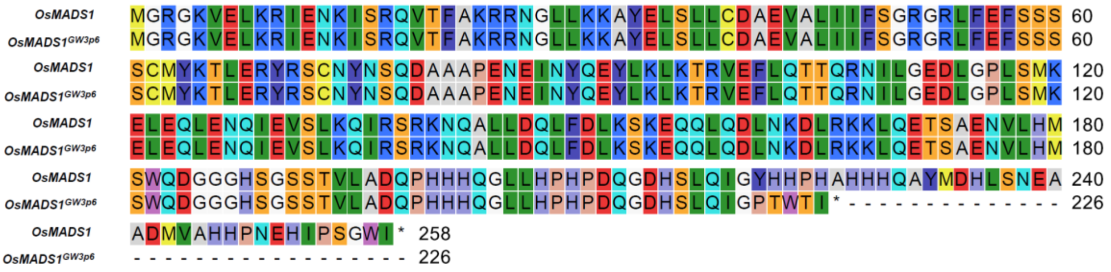

Supplementary Figure 9. Amino acid alignments of *OsMADS1* and *OsMADS1*$^{\boldsymbol{GW3p6}}$. The asterisk represents the termination codon.

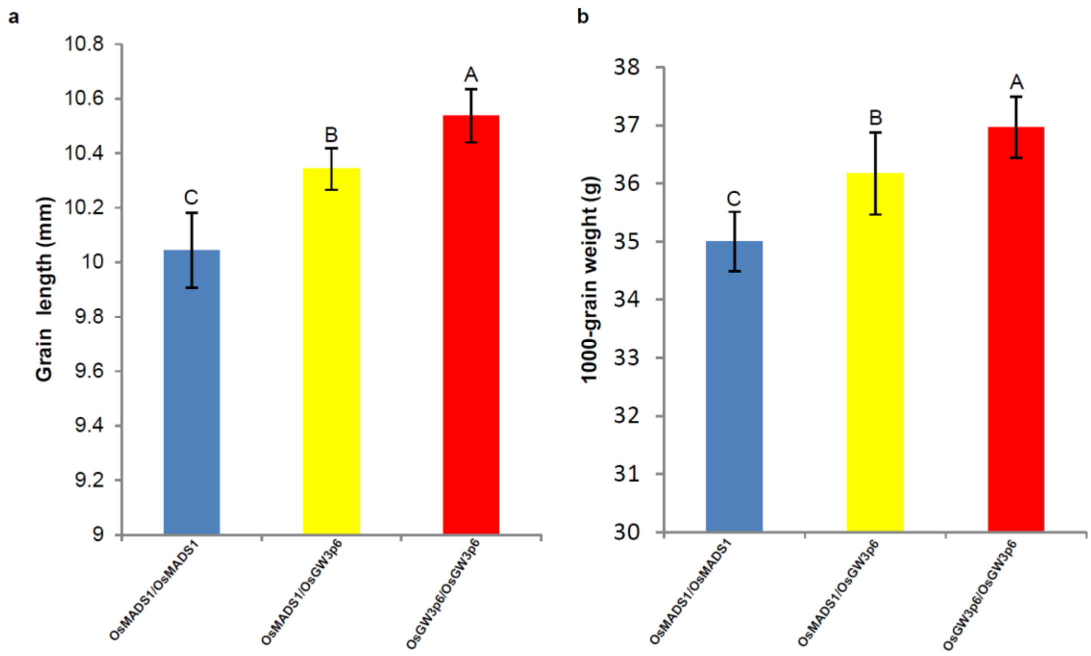

Supplementary Figure 10. The grain weight and grain length of three *OsMADS1* genotypes. (**a, b**) The grain length and 1000-grain weight of three genotypes of *OsMADS1*. (**a**) Blue, red and yellow columns indicate the grain length of three genotypes of *OsMADS1*, respectively (blue, *OsMADS1/ OsMADS1*; red, *OsMADS1*^{GW3p6}/ *OsMADS1*^{GW3p6}; yellow, *OsMADS1/ OsMADS1*^{GW3p6}). (**b**) 1000-grain weight of three *OsMADS1* genotype. Marker CS-92 linked to non-homologous segment is used for genotyping, and the phenotype of 1000-grain weight is counted according three genotypes. Data shown as means ± SD (n = 200). Statistical analyses are performed by Duncan's multiple range tests. Source data of Supplementary Figure 10a and 10b are provided as a Source Data file.

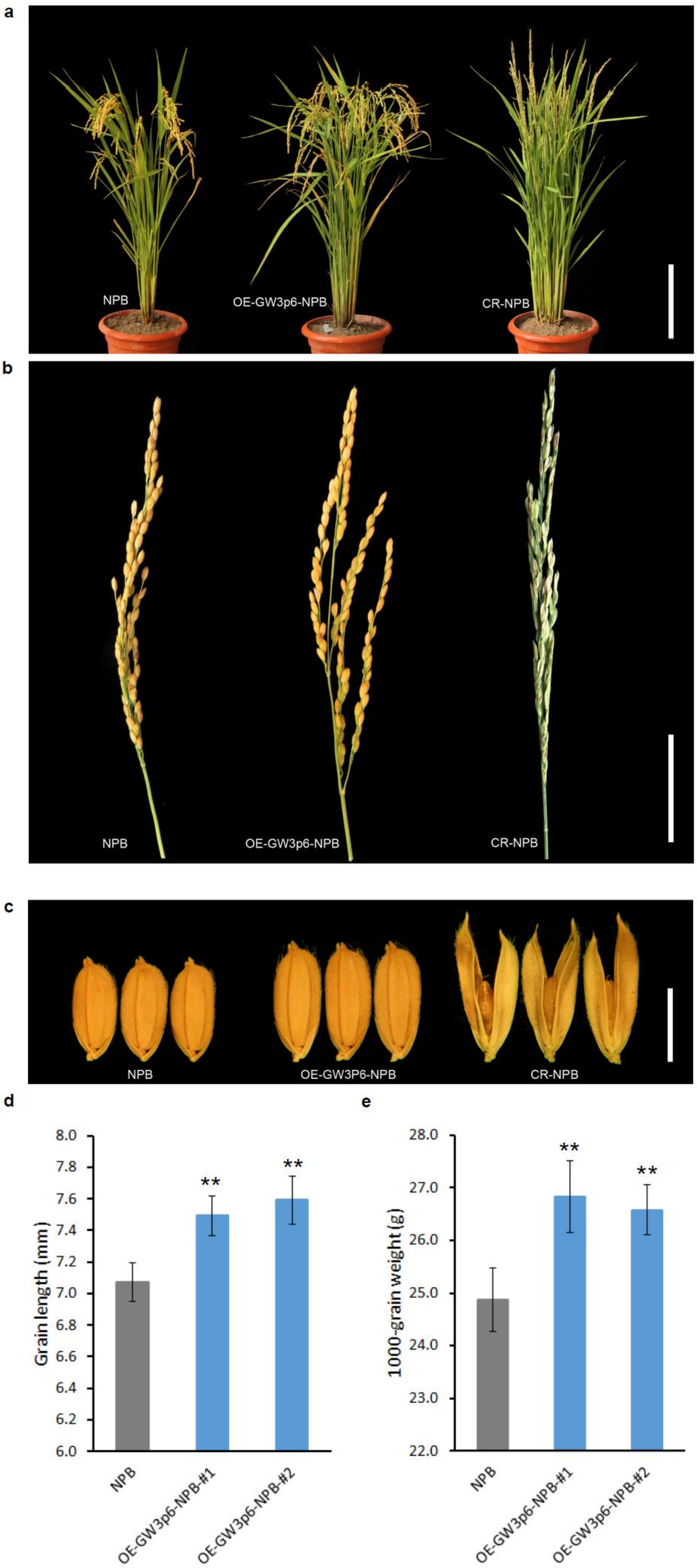

Supplementary Figure 11. The phenotype of transgenic plants and non-transgenic plant NPB. (**a**) The plant phenotype of OE-*GW3p6*-NPB, CR-NPB

and NPB. OE-*GW3p6*-NPB indicates the NPB plants carrying the overexpression construct of *OsMADS1*^{GW3p6}. CR-FH indicates the NPB plants containing missense mutations in C-domain of *OsMADS1*. Transgenic negative NPB is as control plant. Scale bar, 10cm. (**b**) The panicle phenotype of NPB, OE-*GW3p6*-NPB and CR-NPB plants. Scale bar, 5cm. (**c**)The grain shape of NPB, OE-*GW3p6*, CR-NPB plants. Scale bar, 5mm. (**d**) The bar chart of grain length in transgenic plants and NPB plants. n=24 (**e**) The bar chart of 1000-grain weight in transgenic plants and NPB plants. Data in d-e are given as means ± SD; n=24. **significant difference (P<0.01, t-test). Source data of Supplementary Figure 11d and 11e are provided as a Source Data file.

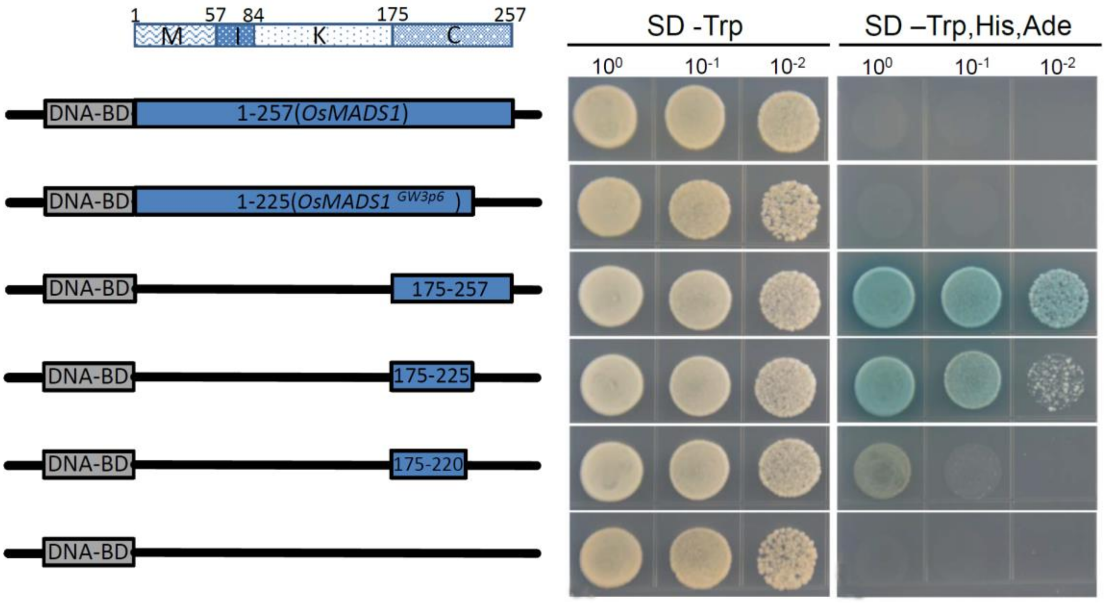

Supplementary Figure 12. Transcriptional activation assay of *OsMADS1* in yeast one-hybrid system. The full-length, C-terminal, and C-terminal incomplete cDNAs of *OsMADS1* were cloned into pGBKT7 such that they were fused to the GAL4 DNA-binding domain. 1-257（*OsMADS1*）indicated the full-length cDNA of *OsMADS1* from FH. 1-225 (*OsMADS1*^{GW3p6}) indicated the full-length cDNA of *OsMADS1*^{GW3p6} from GZ. 175-257 (*OsMADS1*-C Domain) indicated the deletion between amino acids175 and 257, 175-225(*OsMADS1*^{GW3p6}-C Domain) indicated the deletion between amino acids 175 and 225. 175-220 represented the same amino acids between *OsMADS1* and *OsMADS1*^{GW3p6}. Numbers indicated the amino acids of the truncations or deletions of *OsMADS1*.

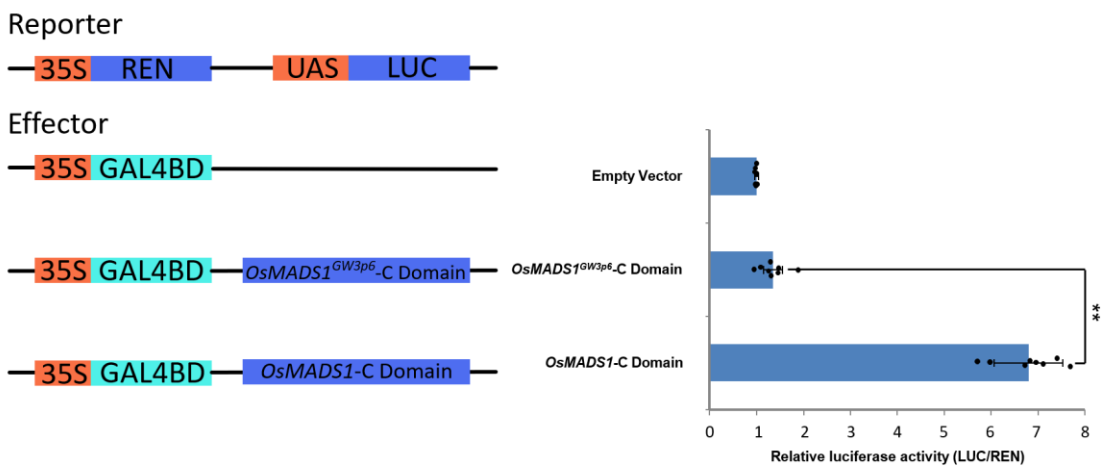

Supplementary Figure 13. The transactivation activity of *OsMADS1* in transient transcriptional activity assays. The effect of the *OsMADS1* C-Domain induced transactivation activity in transient transcriptional activity assays. Schematic diagram depicting the constructs used in transient expression assays in rice protoplast. The reporter vector was co-transfected with effector constructs with different C Domains of *OsMADS1* and an empty effect construct. Relative luciferase activity was monitored in rice culm protoplasts co-transfected with the reporter and different effect constructs. The data are presented as mean ±SD, with three biological replicates. n=8, **significant difference (P<0.01, t-test). Source data are provided as a Source Data file.

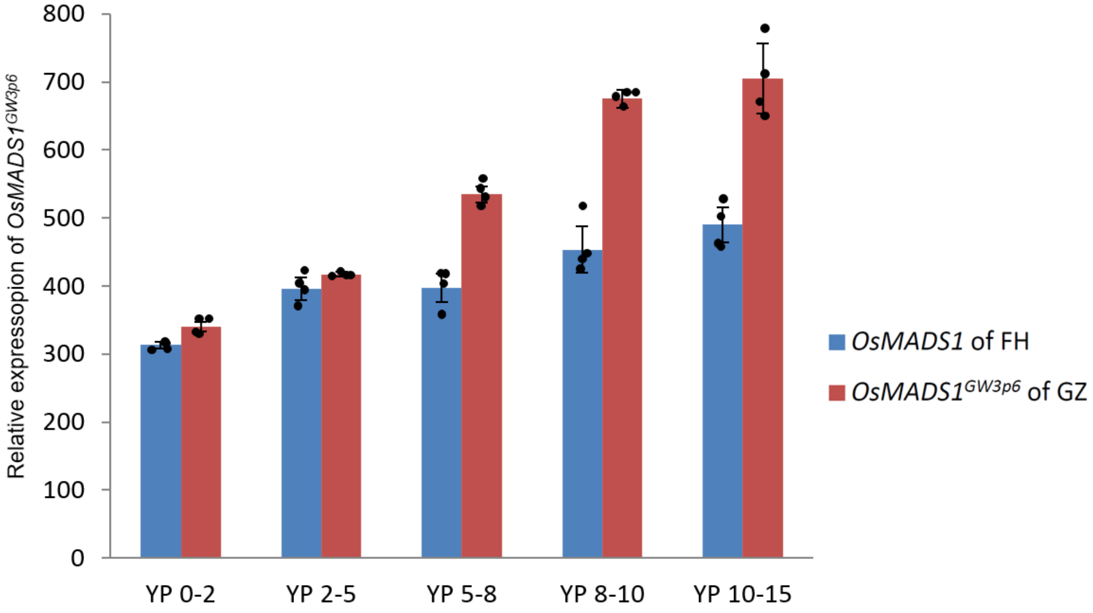

Supplementary Figure 14. The relative expression of *OsMADS1* and *OsMADS1*^{GW3p6} in rice young panicle. The relative expression of *OsMADS1* and *OsMADS1*^{GW3p6} in rice young panicle. The expression levels are shown as relative number of copies per 1,000 copies of rice *Ubiquitin 5*. YP 0-2, YP 2-5, YP 5-8, YP 8-10, YP 10-15 indicate the young panicle of 0-2cm, young panicle of 2-5cm, young panicle of 5-8cm, young panicle of 8-10cm and young panicle of 10-15cm, respectively. Data are given as mean ± SD, n=4. Source data are provided as a Source Data file.

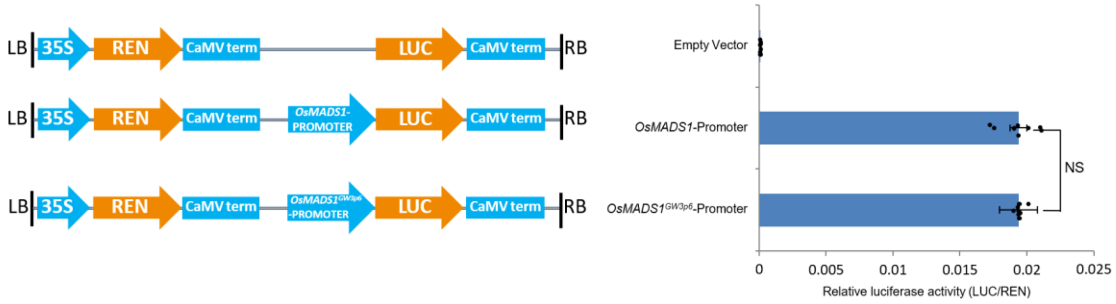

Supplementary Figure 15. Transient expression assay of promoter activity in dual luciferase assay system. Schematic diagram depicting the constructs used in transient expression assays in rice protoplasts. The constructs with about 3.8-kb upstream promoter fragments of *OsMADS1* and *OsMADS1*^{GW3p6} were transfected in rice protoplasts, respectively. Relative luciferase activity was monitored in rice culm protoplasts by luminometer. The empty vector was used as negative control. The data are presented as mean ± SD; n=8. NS not significant difference (t-test). Source data are provided as a Source Data file.

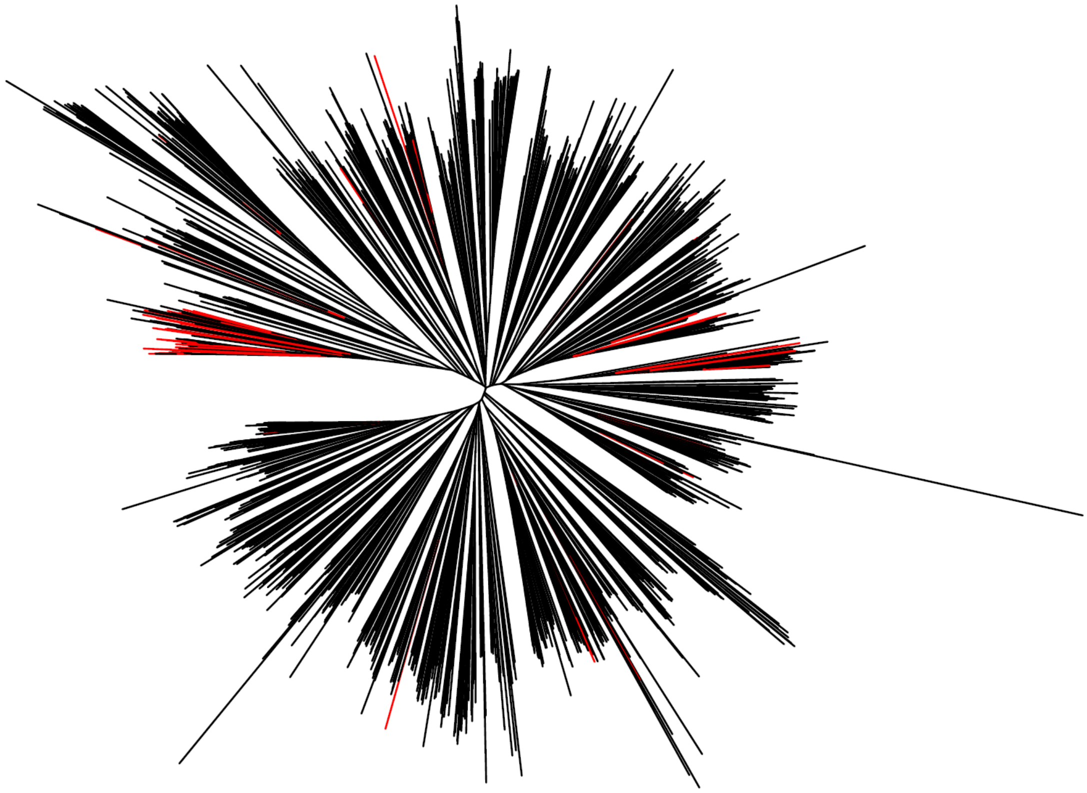

Supplementary Figure 16. The distribution of *OsMADS1*^{GW3p6} in NJ tree of 1,439 indica-indica hybrids. NJ tree of 1,439 indica-indica hybrids constructed from simple matching distances of whole-genome SNPs. The haplotypes of *OsMADS1* were classified into two categories: *OsMADS1* and *OsMADS1*^{GW3p6}, the iTOL (version:4.2.4) was used to display and annotate the phylogenic tree, the red annotation lines indicated the haplotype of *OsMADS1*^{GW3p6}.

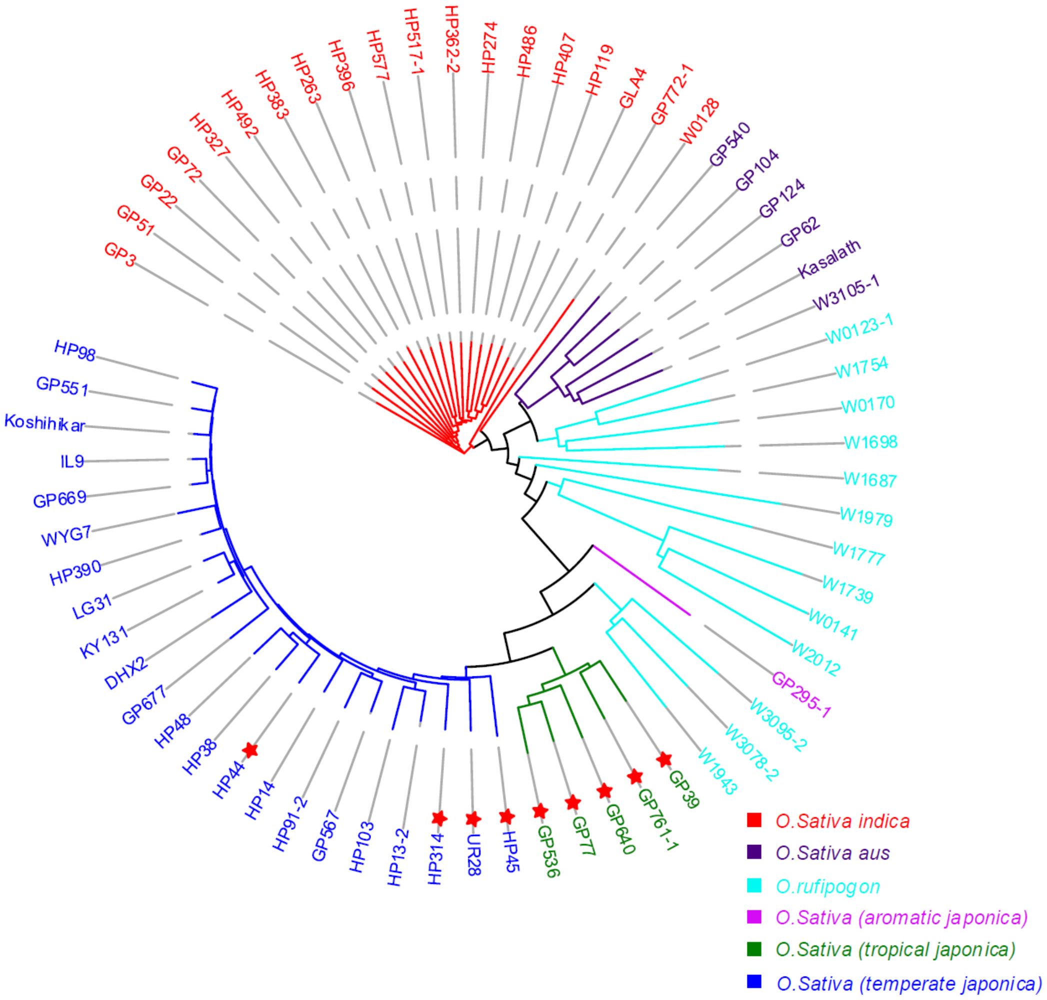

Supplementary Figure 17. The distribution of *OsMADS1*^{GW3p6} in 66 rice accessions. Neighbor-joining tree of the 66 rice accessions was produced by whole-genome data, and accessions within different groups were indicated by different colors. Non-homologous segments of *OsMADS1* as molecular markers to find the haplotype distribution in rice accessions. The red pentagram indicated the accessions contain *OsMADS1*^{GW3p6}.

Supplementary Table 1. **Group information of four agronomic traits**[a]

Trait	Population	Pool	Phenotype	Pool size[b]	Percentage[c]
Plant height	1	1	<79cm	123	33.88%
		2	84-90cm	120	33.06%
		3	>95cm	120	33.06%
	2	1	<59cm	124	32.38%
		2	60-69cm	134	34.99%
		3	>70cm	125	32.64%
	3	1	<65cm	120	50%
		2	70-85cm	120	50%
Heading date	1	1	Before 2/29	103	18.01%
		2	2/29-3/6	102	17.83%
		3	3/7-3/12	118	20.63%
		4	3/13-3/19	125	21.85%
		5	After3/19	124	21.68%
	4	1	Before 2/29	121	19%
		2	2/29-3/6	165	25.9%
		3	3/7-3/12	121	19%
		4	3/13-3/19	112	17.58%
		5	After3/19	118	18.52%
Tiller angle	2	1	Small tiller angle	122	49.59%
		2	Large tiller angle	124	50.41%
	3	1	Small tiller angle	110	50%
		2	Large tiller angle	110	50%
Flag leaf angle	3	1	Small flag angle	114	50%
		2	Large flag angle	114	50%

[a] Phenotypic investigation was conducted in spring of 2017.

[b] Pool size indicate the number of individuals with similar phenotype in pool.

[c] The percentage indicate the ratio of the number of individuals in each pool to the total number of individuals.

Supplementary Table 2. Panicle number of different plants harboring heterosis genes

(I) Genotype	No. of plants	Panicle number per plant (n) [a,b]	(J) Genotype	Mean differences (I-J)	Standard error	Significance (*p* value)[c]
FH	70	7.200±1.566	GLY-676	-7.667	0.478	3.653×10^{-14}
			NIL-FH::*GW3p6*	0.014	0.284	1.000
			GW3p6+PN3q23	-1.114	0.284	6.755×10^{-4}
NIL-FH::*GW3p6*	70	7.186±1.544	GLY-676	-7.681	0.478	3.653×10^{-14}
			FH	-0.014	0.284	1.000
			GW3p6+PN3q23	-1.129	0.284	5.568×10^{-4}
GW3p6+PN3q23	70	8.314±1.975	GLY-676	-6.552	0.478	3.653×10^{-14}
			FH	1.114	0.284	6.755×10^{-4}
			NIL-FH::*GW3p6*	1.129	0.284	5.568×10^{-4}
GLY-676	15	14.867±1.246	FH	7.667	0.478	3.653×10^{-14}
			NIL-FH::*GW3p6*	7.681	0.478	3.653×10^{-14}
			GW3p6+PN3q23	6.552	0.478	3.653×10^{-14}

[a] All data is given as means ± SD.

[b] Data for the grain yield per plant are based on a field experiment using a randomized complete block design. The panicle number is counted one week before harvesting.

[c] *P* value produced by the Tukey's HSD.

[I,J] Panicle number of plants with different genotype.

Supplementary Table 3. The primers used for fine mapping and sequencing assays

Primers	Location (IRGSP4.0)	Forward sequence (5'-3')	Reverse sequence (5'-3')	Type
MP-10	Chr3-5501404-C-T	CAGCTGCAGACCACGTTCAATGAC	TTAGAAGGTACCGAGGCTTAGATCG	SNP
MP-11	Chr3-5600588-G-T	CCAACCGTGCAACATGTACAGTAGC	ATAGATGACATTAGATAGGGATCG	SNP
MP-12	Chr3-5900895-A-G	CACCTCGATGGTTCCACTGCTGTTG	TAGCATCATTTAAAGTAGTATAGC	SNP
MP-13	Chr3-6005440-A-T	GTACGGCCATGTCCATGGCTACGAG	AACCAATCACAACTATCCACTATC	SNP
MP-15	Chr3-6201765-T-C	GCTTGAGTGTCTGACACGATAGTCG	GCAACGCACGGGCACCCTACTAG	SNP
MP-16	Chr3-6300803-A-G	GAACTGCAGCATGGTACAGCCCATG	CTTCATAAAGGACCTACAGGTTCC	SNP
MP-17	Chr3-6409603-T-C	ACTTGGATGGATACAATTCTCAAGC	GACAAGGTTTGCTTTCTACAAACC	SNP
MP-18	Chr3-6500402-G-C	GATGGCTGATATAGGGCCATGAG	CATTACAACTTTCTGTGAGCAGC	SNP
MP-20	Chr3-7001605-G-A	GCAAGGACGAAAGCGACTAGTTTAGG	CTCAACTCAACCACTAATTCGAACAG	SNP
MP-66	Chr3-5945088-C-T	TGGACGAAGACCAGTGCTCCTTCG	ACCCTTGAATCTTAGCATTTGGCC	SNP
MP-67	Chr3-5975780-T-A	CGTAATACCAAGGTAGCTTGTCTG	ATTTACAGCTTCTTTCTATTGCCCG	SNP
MP-68	Chr3-6033720-G-A	CTACCAGGGTTTGCAGAACCTATGGC	GTTGAGCTACGTATGTTACCATCGTG	SNP
MP-69	Chr3-6050905-T-C	CCTACCGAACAACCTCAACACCTCAC	TATTACATCACCTTTCAATGCATGAG	SNP
MP-71	Chr3-6136056-A-G	CGCACACGCGCATGCACGGACATGAC	GCTGCAGTTAGGGCCTGGGTAGATCG	SNP
MP-72	Chr3-6158291-C-T	CACCAACAGTACGAAAGCCCAATGTTCG	CAAACCAGTGTCAAAGCTACAGATGTAC	SNP
MP-73	Chr3-6183473-G-T	GGTTTGTCGTACGTGGCACCCGTC	GAGTATACCGCTTCGACACAGGTC	SNP
MP-76	Chr3-6280171-A-G	ATGGAGGACGTAAGGTGGCAAGTACG	GCGCACTGCTTGCCGCATGTCGCGC	SNP
MP-79	Chr3-6429638-C-A	CTTCCATCTGCTCGTATCAGGTTGTC	GGCCAAGGGCACGATCTTTGCGTCTG	SNP
MP-80	Chr3-6481007-G-A	TCTACGTTTTCTACCGGTCTCCGGTG	GGTATGAGTGCGTCGCCACTACTACC	SNP
MP-81	Chr3-6752348-G-T	CTGTAGCGAGCCCATCGGATCGGAG	CACCTGCCATTTCGGTGATGAACTTC	SNP
MP-82	Chr3-7102085-T-G	CCAGCTAGCAAACGATCTATCTAGAC	GCCTCAACTCGGATATGTAATTAAC	SNP
MP-87	Chr3-6052189-G-A	GTAGGAGAGGTACGGTAATTCCGTG	GAAACTAAAGATCCTCGTCTCCTGG	SNP
MP-88	Chr3-6053200-T-G	ATTTGATTTGTGTGTAAGTAACTTTGG	CACAGCCTTAGAGTATACCGAATCACTG	SNP
MP-89	Chr3-6055559-A-T	CCTAGTCATGTGTGGCGTGCCCAC	GTACTTGTTCAGAGCGCCAACGCTGC	SNP
MP-90	Chr3-6058017-A-T	GCTTGTGATTCAATGACGATGCCAATC	TCACAAATCACTTCACTATTTCAACC	SNP
MP-91	Chr3-6060346-T-C	GGTATGACAGTGCACCTGTCCTGGC	ACTGGCTAGCTAGACACGTATTGGC	SNP
MP-92	Chr3-6064242-C-G	CATCGCTAGCGAGAGCCACTAGTG	GTATCTCTGCCGACATATGATATGG	SNP
MP-94	Chr3-6069500-G-A	GTGGTAGAATGTTCTACTCAGCTGG	GGTCACGGTGTCCATCAGCCTCTTG	SNP
MP-95	Chr3-6070768-C-T	GATCCATGCATGGCAAGCGTTTGAG	AGTGTGCATATAGTTGCTTTGCTAC	SNP
MP-97	Chr3-6074580-A-G	CACCACACATGCATTATTATCCAATG	TTACTTCATCGATGATATATCGAAG	SNP
MP-98	Chr3-6077193-C-T	CATCACATATGTACATGACTACTTG	GTTCAGATCTAGCTTGGATCCGATC	SNP
MP-99	Chr3-6078639-G-T	ATGCATCGATCTGAGATATTCTGG	CACTCCAACATACATCATGGAACG	SNP
MP-100	Chr3-6083050-T-G	CTGGAGCTGCTGCATCCTGTGAGTTG	CCTTCTGTAGTAATTTCTATATTAG	SNP
MP-101	Chr3-6086698-A-T	CGATGCATGCAGGACTAGCTCTG	GAGCTTCAGACGTGTCATATCAAGC	SNP
MP-102	Chr3-6089578-G-A	CTGTCTCTCACTCGCTTCAAGTC	TAGTCGGATAAGATAGTCACCGAC	SNP
MP-104	Chr3-6098500-T-A	CGAATGGAGTATCATGATAAAGTGG	GTCATCGCATATCATATCTCACTAG	SNP
CS-92	Chr3-6080311—6080756	CGCCCAATAATCTAGATCAAATTGG	GATACAGACATACAGTACGTGCATC	InDel

Note: The file includes the information of primers used for positional cloning and genotyping by Sanger sequencing.

Supplemental Table 4. Primers used for constructing NILs

Primers	Location (IRGSP4.0)	Forward sequence (5'-3')	Reverse sequence (5'-3')	Type
MP-1	Chr2-501146-T-A	GACTGATTCTCCTCATACTTCCTGG	AGCCCTGGAGCCTTATCATTCATCC	SNP
MP-2	Chr2-2002748-A-G	GCAAGCTCATGCTCCGTGGCTAC	TGACCGTGCAGTTGCGCAGTAG	SNP
MP-3	Chr2-4000866-C-T	CGTGATTAGCGAAATTGTCGCTG	TGCACCATTGTACCCACCTGGAC	SNP
MP-4	Chr2-4501186-A-G	GTATCCGGCCCATTATCATCCACCC	AAGGGAATGAGCTTGGCTTCTGCTC	SNP
MP-5	Chr2-5004806-C-T	TGGTCGTTAGCTGGTGTTGCTTCCC	GGAATGGATGACCAATCAAAGGAGG	SNP
MP-6	Chr2-6404258-C-T	GACTACTACTGCAACCCCGTAAG	GTCAATTCCAAGCGCTGCTTACTG	SNP
MP-7	Chr2-7032627-A-G	GATACTCTAATCTCACACCTAGTG	GGATAGTGAGAATAGAACGGTGG	SNP
MP-8	Chr2-8012323-T-A	CATAAGTTGTCGTTCAGGGGAAATG	GGCATATTTACGTTGCGCATGGAC	SNP
MP-21	Chr3-8007764-A-G	CTAATGCTGAACCATGGACCGGTTG	CAAAGACACCATATCATCAGGATGACG	SNP
MP-22	Chr3-9000361-T-C	CCAGAGCTTCTTGCGCCTATCATG	TCTCCCTCTAATCTATATTTCCAAC	SNP
MP-24	Chr3-25045960-A-G	GTCCTACCTACTGACATTCAAGCAAG	CGTGTGAACAAAAAGATAGGAGATGTGCTG	SNP
MP-25	Chr4-33001248-A-G	GAACTTTTAGCCAGAAATCATAGTC	CTCAACATTGTAGGCACTGGAGGAC	SNP
MP-26	Chr4-34000332-A-T	CTCTCCTGAATAGCAGCTGCGAGAG	GGCAAGGACTTGGCAAGAAATACAAG	SNP
MP-27	Chr4-34200034-G-A	CGATTCAGCTCGCTACGTGGCTAC	TGAGTACATGCATCTTATTATGCTC	SNP
MP-28	Chr4-34502168-T-G	CATGAGGCGAACTCCCTTAAGGG	CAAAGTGAGCCTGGAGTATACAG	SNP
MP-29	Chr4-35000072-T-C	GTCGATCACGAAGCTTCAGGTTAGC	TCAGAGGAAGGACCATGTAGCTTGC	SNP
MP-32	Chr5-7000089-G-A	CATGCGCTCATGGATGCAAGCTACAGC	AGGTCAAAACCACAATTGAACGAATGGAG	SNP
MP-33	Chr5-7504273-G-A	CAACGAGCTGAAAACAATCCAAGGGC	CTCAACCACTCCAAGTGGCTGGACCTCTC	SNP
MP-34	Chr5-22008527-T-C	CGAACGAACAGGTCATTATAAGAGAG	AGTTCCATCGTCCTATTATCGGC	SNP
MP-38	Chr6-32032511-G-A	CAGGGTATTGCACTGATAATTACTG	AATGCAAATGCAACTAACAGCCCAGGTAG	SNP
MP-40	Chr7-7000016-G-A	CATTGCGGGCTTTGTGAGTGTGACGG	GAGGTGTTCGATCTTGGGACTTGAG	SNP
MP-41	Chr7-8000285-C-T	GATCAACCTATTTGCTGTCGTTTGGATC	CTGCTGATTTGGCTATCAGTGGTAACTG	SNP
MP-46	Chr7-20006362-G-A	GAGGAAGTACGTTACAGAGAC	CCGAGAGCTAATATAAGCTAACACG	SNP
MP-49	Chr8-27900213-C-T	CTGCCCACGAGTAGTTTGATGGGTG	GATTTGTGTGGCAAGTGTGCTCTTAAC	SNP
MP-52	Chr11-25000170-G-A	CGTTCTTCGGTGAAGTAGATGGCCG	CACGTGGCAGGTGTTTACAGGGTC	SNP
MP-53	Chr6-21896540-A-C	CAGTAGGCGGCAAACAAGGGTGAGAC	CAAGTAGTCAAGCCGACATGTTAG	SNP
MP-54	Chr6-22195060-T-C	CTTTTCGCCATGTTGCAGGGACAATATG	CACTCTCCTGACAACGTAGAAGGAC	SNP
MP-55	Chr6-22200098-T-C	CCAGGCCTGAGTACTAATTGGGTTTG	GTGGCGGTGGACCACTTAAGCAAAAC	SNP
MP-56	Chr6-22220737-C-T	TATATTACAAGGGAAGATCCGAGTAC	ATCTCAGGGTTAGTCTGACATCAACTGC	SNP
MP-57	Chr6-22276503-G-A	CTCGCTTGATCCCATGATAATCAG	GAAGTTGCACACCTGCAGCACGGAATG	SNP
MP-60	Chr6-22489827-A-G	CAGGGTCTCCACCGTCTATTGCTG	CATGAAGTGTATAACTAATACTGCTG	SNP
MP-105	Chr2-1008525-A-T	CAATAAACGTTGTATATGTGCTCGG	GCTCCATCTCTCCAGGAGTGCAGGC	SNP
MP-106	Chr2-1013682-T-C	CAAGTAGTTGTCTAAAATAGCGACGG	CTCTCTCACCTCTCTCACTATGTTTC	SNP
MP-110	Chr3-15029270-A-G	GTTAATTACTAAGAGCTAGTCAAGG	TTGTTCCGTCTCGTATTCGCTCCG	SNP
MP-111	Chr3-22525107-A-G	AGTTTATTTTCTAGCTATCATTAGAGC	TTATCTTGTGCACTTGCACCTGAACTA	SNP
MP-112	Chr4-32998240-A-G	ATATGATCAAGATGTCTATCTAGACT	TATCCTGTCTGTCCTGGATCCCATC	SNP
MP-113	Chr4-34020910-G-A	GCATTTAGGCACGCACCAAATCCTTG	TGTCTGTCAGTGAGTGTTGCAGTGC	SNP

MP-114	Chr4-35122560-A-G	CTATATTACTCCTTGTGGTAGCATGTC	TTGCCGACTGTCACGCAATTAATAC	SNP
MP-115	Chr5-1000045-A-T	GGAGTACATAGTGGTATTCGTACGG	CTGCCATGTTGGTAGAAACCACTTTC	SNP
MP-116	Chr5-2500205-A-T	TTCTCCTCAGCGGCAATATTGTTCAC	TCAGCCTGGAGGTAAAACTCTCTAC	SNP
MP-117	Chr7-20262928-G-C	GAATTAATCAAGCTTACCGGAAAGG	CCGTCCATCTCGCATCCCACGGTCC	SNP
MP-119	Chr9-4819193-C-A	CACAGCATTATGTAAGTGTTAGATCC	AACATGAAACATTGCTTTCATAAGT	SNP
MP-120	Chr9-7412698-C-T	CCTATAATTGATAGGAATTCCTTAGC	CGTGCTTACGTCAGTACCATTGTCTC	SNP
MP-121	Chr11-24841592-A-G	AACTAACTTCTACAAGGTTATTATGC	ACGTAGACCCTCTAGCCCACACTATC	SNP
MP-124	Chr4-32174635-T-G	GGTGGTTGGCGATGCCTTGTTAATCT	GTACTCCTTCCAGGAAGTTTCAAGC	SNP
MP-125	Chr5-1739475-T-C	GATAAATGTATGGTAATTTCTGGAGG	TACTTAATTAGCTAGTTATATCTTCC	SNP
MP-128	Chr9-6660351-C-T	ATCCATTATCATGGGATACGTTTCC	GCGATGTCCTCTCACCCGATGGGTT	SNP
MP-131	Chr3-23000404-C-T	GCGTGTCTGACAGGGTTGAAATCACC	CGACGGTTGATTGCTGGATTAGCTGTC	SNP
MP-133	Chr3-23256419-T-C	GTTGCGTGCGTATGAGAAGTCGC	CCCATATATTTAGGGTGGAGCCC	SNP
MP-134	Chr3-2200046-A-C	GACCTCTACGGTAGGGTGCATTTCTCAG	CCTCATAAGGTAGATCAATAAGCTATCTC	SNP
MP-135	Chr8-3500770-A-G	CTTCGTTGGCTATACGATGGTCG	GACGATCAGTACTCAAACGATAG	SNP
MP-136	Chr8-28033248-C-T	CTGTGCCTCTCCCTCGACACAAGC	CCCATGACACGGTATGCATTGCTAC	SNP
MP-137	Chr3-23000404-C-T	GCAGCTCATGAATATTACAC	CGCGAAAAACGAGGGAGATG	SNP
MP-138	Chr3-23256419-T-C	GGCAACCCTAGTCATCTTTAG	GATGTGATGAAAATGTTGGAAG	SNP
MP-140	chr3-16102974-16103034	AATAGGGACAGTTGTCTTACGCC	GTTGTTGTGCGTTCATATGTACAG	InDel
MP-141	chr3-16395927-16399116	gtttaactcctgatttggaacgg	ctacatcattggtgagatatagc	InDel
MP-144	chr3-20999753-20999809	GAAGAAGAAGTACGCGCGTAG	CTAGTCAGGTGTCAGGATGTC	InDel
MP-145	chr3-21954584-21954617	GGGAGATAATAAGGGACTTGG	TTAACCGTGTcatgtacggctcc	InDel
MP-149	chr3-22772282-22772320	GTTTTGTTTGACTCGCTGACGC	CTAGATCGTAAGGTCTAGATCTGC	InDel
MP-152	chr3-23058536-23058581	GCCAATCACTTATACATTACAGC	CGGGTGGTTTCATATCCTCTATAG	InDel
MP-153	chr3-23295139-23295176	GACAGAGGATGTGAATCTCGTGGC	ccatgtagccagtcatgtgtggaag	InDel
MP-155	chr3-23564746-23564779	GAATTTAGCATGGTTGTTAGGGAC	ACTACGGTATGTCCTATCTGTTC	InDel
MP-156	chr3-23785070-23785112	gacaatatactagcgtggatgtg	cttgagaattgaatgttcgagacc	InDel
MP-158	chr3-24498261-24498332	GTAAGTAGATTCCATTCCTTGTAG	CAGAAAATACCTGATGaggcggc	InDel
MP-159	chr3-24512689-24512723	CTTATCTCTAATCCTCTCTTCCAC	AGCCTCTTTTGCAACGTGGATCG	InDel
MP-177	chr3-21938614-21938736	GGTAGAGACCCTTAAGCCTTAAAGC	ctccaagaaatttatattaaattc	InDel
MP-178	chr3-21954525-21954727	GCAAGTTAGAATTCTAGCGGATC	GTGAATGAGACCCCTTTAAATTGC	InDel
MP-182	chr3-24498111-24498332	CTGACAGTTGAGATGGCACGTAAG	CAGAAAATACCTGATGaggcggcg	InDel
MP-191	chr11-25754636-25754891	gagaaatgctcctgacttttgg	GCTCACATCACAGCAAAAACTCTG	InDel
MP-192	chr2-894370-894695	GTTCATCACTGATGTGAGATAGAAG	CTGTTATGTATGATCTCTGGGAGTG	InDel
MP-193	chr2-481554-481770	GTGAGATGCAACTTatcaaagtcg	catgtctaaatagaactcttatcgg	InDel
MP-195	chr3-20999608-20999875	GGAAGAAGAAGTACGCGCGTAGac	TCTTCTGCTCATGTGGTCTATAAC	InDel
MP-196	chr5-2238196-2238360	ATCCCTCTTTAGGGTGGGAGACATGC	CTCCTCTAGTAACCTACGTGCTCCTG	InDel
MP-198	chr5-22326658-22326880	ctaaacaggcccTTTCACGAGCATC	cctagttaaaagagccacatagacc	InDel
MP-199	chr5-22512679-22513036	GTTCAACATTTCAATCACCTGCTGTC	ctaagAGCTCGTATCAGACTTAACG	InDel
MP-204	chr8-26327072-26327368	GCCTATTCACTTTACTGCCCATG	GTCGGTCAAAGTTGTCAGCAGTG	InDel
MP-210	chr5-4067804-4068055	GAGGTTTAGCGAGTTGTGAAATTAAC	CAACAATATAATTTATGGGGTGCACAG	InDel

MP-211	chr5-6479290-6479588	GCGATCGATCGAGCGTGTTTG	gacataagtagtgtactgtagc	InDel
MP-212	chr5-6574494-6574697	CACAGTATAAGGGAAAGCAGACTGC	AATAACAGTTCTTAATACAGGAGCC	InDel
MP-217	chr4-30399695-30399986	GTACTTGCCAAGTTGCAACGGACG	GCTATCGACCAAGCGTTAATATAG	InDel
MP-221	Chr3-21014407-C-T	GCTGACTACACGACTATACCGATGC	CGAACCTCTTAGCGTGGCCGCTAG	SNP
MP-231	Chr10-12406000-A-G	GTGAATAAGGACGCGGGTCATTAGCC	GCGCGCGGAGTCAGGAGTGCGTGC	SNP
MP-232	Chr10-13067495-G-A	GAAGGAAGAACCGATTCAGTACG	GGATTTTCTCAATCACCTGCTCTGAC	SNP
MP-235	Chr3-26004692-C-T	TTAGTTGTCCATCGACCTCTGGTAGG	CTGTCACCGAGGGCACCAAATTTTTGC	SNP
MP-241	Chr3-26065973-T-C	CAGAGAGGAGGTCTAATTCAACCCTT	CTCTGGCGCCAGTCCTCCTGAACAG	SNP
MP-248	Chr3-22427796-T-G	CAACCTTATCAAGCAACCGTGTTG	GTTGAGAAACCACCAATAGAGATTG	SNP

Note: The file includes the information of primers used for genotyping to construct NILs, and the SNP markers used for Sanger sequencing, InDel markers used for genotyping by gel electrophoresis.

Supplementary Table 5. Primers used for real-time RT-PCR and plasmid construction

Primer	Forward sequence (5'-3')	Reverse sequence (5'-3')	Purpose
OsMADS1-RT	ATCCCATCCGGCTGGATATGAT	ATTACTGGATTACAGGACACTG	real-time PCR for *OsMADS1*
Ubi-RT	ACCACTTCGACCGCCACTACT	ACGCCTAAGCCTGCTGGTT	real-time PCR as internal control
OE-*MADS1*	TTTGGTGTTACTTCTGCGGCGCGCCAT GGGGAGGGGGAAGGTGGAGCTGAA	AGGAACATCGTATGGGTAAGGTACCGG TATCCAGCCGGATGGGATGTGTTC	*OsMADS1*-OE plasmid construction
OE-*GW3p6*	TTTGGTGTTACTTCTGCGGCGCGCCAT GGGGAGGGGGAAGGTGGAGCTGAA	AGGAACATCGTATGGGTAAGGTACCGG GATGGTCCATGTAGGCCCAATCTGCA	*OsGW3p6*-OE plasmid construction
CR-*OsMADS1*	GGCATGAACACATCCCATCCGGC	AAACGCCGGATGGGATGTGTTCAT	Crispr-*OsMADS1* plasmid construction
pGBK-FL-*MADS1*	CGGAATTCATGGGGAGGGGGAAGGTG GAGCTGA	CGGGATCCTATCCAGCCGGATGGGA TGTGT	Full length *OsMADS1*-BD plasmid construction
pGBK-FL-*GW3p6*	CGGAATTCATGGGGAGGGGGAAGGTG GAGCTGA	CGGGATCCGATGGTCCATGTAGGCC CAATCTG	Full length *OsGW3p6*-BD plasmid construction
pGBK-CD-*MADS1*	CGGAATTCTTACAGGAAACCAGTGCAG AGAATG	CGGGATCCTATCCAGCCGGATGGGA TGTGT	C Domain of *OsMADS1*-BD plasmid construction
pGBK-CD-*GW3p6*	CGGAATTCTTACAGGAAACCAGTGCAG AGAATG	CGGGATCCGATGGTCCATGTAGGCC CAATCTG	C Domain of *OsGW3p6*-BD plasmid construction
pGreenII-FH&GZ-Promoter	CGAGGTCGACGGTATCGATAAGCTT AATGTTACTCTCCGTCCCAGTGCC	CCGCTCTAGAACTAGTGGATCC CTTCTTCCTCCTCCTCTCCTCTCTCTC	pGreenII-FH&GZ-Promoter plasmid construction

Note: The file includes the detail information of primes used for quantitative PCR and plasmid construction of functional assays.

Supplementary Note 1

Evaluating the effect of experimental variables

From the above validation experiments, our method showed pretty well performances in detecting related genetic intervals of QTLs. Furthermore, we preferred to explore the whole procedure of our approach in depth, especially the influence on results when changing experimental variables. For this purpose, we conducted a computer simulation experiment to examine five aspects (1) the percentage of individuals in each group we selected, i.e., pool size, (2) the read depth, (3) the number of bulks, (4) the effects of misclassification, (5) different statistical algorithm.

We postulated that total number of F_2 generations is 500 and simulated 1000000 SNPs between two parental genomes according to practical experiment. In the first step, we evaluated the errors of false positive while changing the pool size (N), the read depth (G) and the number of bulks (B). For an unlinked SNP, if we assumed that the depth was G, both the number of reference alleles and alternate alleles would fluctuate around G/2. In Supplementary Fig. 5a, we fixed depth G as 100×, changing N from 20 to 100 and B from 2 to 5. While in Supplementary Fig. 5b, we changed G from 20 to 100 and the number of bulks from 2 to 5 with the fixed N. After performing statistical test, we calculated the 99% cutoff value of –ln(*p-value*) under null hypothesis that there is no QTL. The results illustrated that the pool size significant affects errors of false positive, and the number of bulks and depth do not influence much on the results.

In the next step, we evaluated the power of detecting the QTL if misclassification (Mis) happened, assuming all in the case of complete dominance. In these circumstances, we set the value of QTL effect as 75% and supposed that p% of individuals in a certain bulk were not correctly graded and classified. We considered two circumstances here, 10% and 20% of the certain bulk were misclassified in the

situation of two-bulks, and tried to figure out how to improve the accuracy if the misclassification occurred. Evidently, the more wrong classified individuals led to less truthful results (Supplementary Fig. 5c, 5d). Furthermore, we took the number of bulks into account in the case of 20% misclassification, assessing whether the number of bulks affects the performance in QTL identification. In Supplementary Fig. 5e, we set pool size N =20 as fixed one, exploring the power as depth increases from 20 to 100×. Next, we fixed the depth = 20, assessing the results as N from 20 to 100 (Supplementary Fig. 5f). With the increasing of the read depth and the number of individuals, the power would increase and eventually equaled to 1. The results also revealed that the more bulks we divided, the more likely we were to detect the QTL.

In addition, we made a comparison with three different statistical algorithms (Ridit analysis[1], Kruskal-Wallis test[2], and Chi-square test[3]) to find out the optimal one, and interested in whether there existed differences in processing data among these three statistical tests. From the results by comparing to the known QTLs, we could reach a conclusion here, the former two strategies which are used for ranked data performed better in identifying QTL when compared to Chi-square test[3], and Ridit analysis[1] has the optimum results.

Supplementary References

1. Bross, I.D.J. How to Use Ridit Analysis. *Biometrics* **14**, 18-38 (1958).
2. Kruskal, W. H., & Wallis, W. A. Use of ranks in one-criterion variance analysis. *J. Am. Stat. Assoc.* **47**, 583–621.
3. Pearson, K. X. On the criterion that a given system of deviations from the probable in the case of a correlated system of variables is such that it can be reasonably supposed to have arisen from random sampling. *Lond. Edinb. Dubl. Phil. Mag.* **50**, 157–175 (1900).
