# Supplementary material for: Dissecting a heterotic gene through GradedPool-Seq mapping informs a rice-improvement strategy
Source: Nat Commun. 2019 Jul 5;10:2982. doi: 10.1038/s41467-019-11017-y (PMC6611799; doi:10.1038/s41467-019-11017-y)
Supplement: Supplementary file 3 — Reporting Summary [file 41467_2019_11017_MOESM3_ESM.pdf]

nature research

Corresponding author(s): Bin Han

Last updated by author(s): May 23, 2019

Reporting Summary

Nature Research wishes to improve the reproducibility of the work that we publish. This form provides structure for consistency and transparency in reporting. For further information on Nature Research policies, see Authors & Referees and the Editorial Policy Checklist.

Statistics

For all statistical analyses, confirm that the following items are present in the figure legend, table legend, main text, or Methods section.

n/a	Confirmed	
☐	☒	The exact sample size (*n*) for each experimental group/condition, given as a discrete number and unit of measurement
☐	☒	A statement on whether measurements were taken from distinct samples or whether the same sample was measured repeatedly
☐	☒	The statistical test(s) used AND whether they are one- or two-sided<br>*Only common tests should be described solely by name; describe more complex techniques in the Methods section.*
☒	☐	A description of all covariates tested
☒	☐	A description of any assumptions or corrections, such as tests of normality and adjustment for multiple comparisons
☒	☐	A full description of the statistical parameters including central tendency (e.g. means) or other basic estimates (e.g. regression coefficient) AND variation (e.g. standard deviation) or associated estimates of uncertainty (e.g. confidence intervals)
☐	☒	For null hypothesis testing, the test statistic (e.g. F, t, r) with confidence intervals, effect sizes, degrees of freedom and P value noted<br>*Give P values as exact values whenever suitable.*
☒	☐	For Bayesian analysis, information on the choice of priors and Markov chain Monte Carlo settings
☒	☐	For hierarchical and complex designs, identification of the appropriate level for tests and full reporting of outcomes
☒	☐	Estimates of effect sizes (e.g. Cohen's *d*, Pearson's *r*), indicating how they were calculated

Our web collection on statistics for biologists contains articles on many of the points above.

Software and code

Policy information about availability of computer code

Data collection	All the NGS data collected on the bulked segregant samples were generated by Illumina Hiseq2500 system according to user instruction.
Data analysis	All codes for GradedPool-Seq (GPS) bioinformatic pipeline can be downloaded at http://www.ncgr.ac.cn/software/GradingPoolSeq (Line591 and Line 598).

For manuscripts utilizing custom algorithms or software that are central to the research but not yet described in published literature, software must be made available to editors/reviewers. We strongly encourage code deposition in a community repository (e.g. GitHub). See the Nature Research guidelines for submitting code & software for further information.

Data

Policy information about availability of data

All manuscripts must include a data availability statement. This statement should provide the following information, where applicable:

- Accession codes, unique identifiers, or web links for publicly available datasets
- A list of figures that have associated raw data
- A description of any restrictions on data availability

The sequence data are deposited in the European Nucleotide Archive under accession number PRJEB30329 (Lines 588-589).

Field-specific reporting

Please select the one below that is the best fit for your research. If you are not sure, read the appropriate sections before making your selection.

☒ Life sciences ☐ Behavioural & social sciences ☐ Ecological, evolutionary & environmental sciences

Life sciences study design

All studies must disclose on these points even when the disclosure is negative.

Sample size	Given the results of our simulation and estimating the recombination rate ,a total of more than 1000 bulked samples (351 individuals in low 1000-grain weight pool, 348 individuals in medium 1000-grain weight pool and 347 individuals in high 1000-grain weight pool) out of 1105 F2 individuals were selected based on the graded phenotypic variations in order to fine-map GW3p6 into~400kb genomic region. And the sample size of GPS for other agronomic traits was shown in Supplemental Table1.
Data exclusions	No data was excluded in the analysis.
Replication	Yes. As usual in growth measurements of plants, they were grown in appropriate and similar environmental conditions. For GPS method, an independent experiment using GLY-676 F2 population was conducted. For dissecting the GW3p6, all the agronomic traits were investigated at least 2 times in different area (Shanghai and Sanya). All attempts were successful.
Randomization	We used a large population of F2 individuals, all the F2 individuals were planted randomly under appropriate growth conditions. We measured some traits for each F2 individual,according the phenotypic variation, bulked the relevant samples in one pool uniformly to ensure well-distributed of each sample.
Blinding	Blinding was not possible, because we designed this genetic population to group and analyze every F2 individuals,which ensure the large segregating population for QTL fine-mapping.

Reporting for specific materials, systems and methods

We require information from authors about some types of materials, experimental systems and methods used in many studies. Here, indicate whether each material, system or method listed is relevant to your study. If you are not sure if a list item applies to your research, read the appropriate section before selecting a response.

Materials & experimental systems

n/a	Involved in the study	
☒	☐	Antibodies
☒	☐	Eukaryotic cell lines
☒	☐	Palaeontology
☒	☐	Animals and other organisms
☒	☐	Human research participants
☒	☐	Clinical data

Methods

n/a	Involved in the study	
☒	☐	ChIP-seq
☒	☐	Flow cytometry
☒	☐	MRI-based neuroimaging
