# Supplementary material for: Dissecting a heterotic gene through GradedPool-Seq mapping informs a rice-improvement strategy
Source: Nat Commun. 2019 Jul 5;10:2982. doi: 10.1038/s41467-019-11017-y (PMC6611799; doi:10.1038/s41467-019-11017-y)
Supplement: Supplementary file 4 — Description of Additional Supplementary Files [file 41467_2019_11017_MOESM4_ESM.docx]

**Description of Additional Supplementary Files**

File Name: Supplementary Data 1

Description: Sequence and haplotype analysis of the *OsMADS1* gene in hybrid rice. The InDel marker CS-92 is used to analyze the haplotype of *OsMADS1*, and the haplotypes of *OsMADS1* are classified into two categories: *OsMADS1* and *OsGW3p6*. *OsMADS1*and *OsGW3p6* indicate the homozygous *OsMADS1* and *OsGW3p6* in one hybrid rice variety respectively, *OsMADS1/OsGW3p6* indicates the heterozygous genotype of *OsMADS1*. NA represents not available data due to missing samples.

File Name: Supplementary Data 2

Description: Sequence and haplotype analysis of the *OsMADS1^GW3p6^* gene in parents of hybrid rice. The letter A and S represent male sterile line in three-line hybrid rice and male sterile line in two-line hybrid rice respectively.
