# Supplementary material for: Dissecting a heterotic gene through GradedPool-Seq mapping informs a rice-improvement strategy
Source: Nat Commun. 2019 Jul 5;10:2982. doi: 10.1038/s41467-019-11017-y (PMC6611799; doi:10.1038/s41467-019-11017-y)
Supplement: Supplementary file 2 — Peer Review [file 41467_2019_11017_MOESM2_ESM.pdf]

Reviewers' comments:

Reviewer #1 (Remarks to the Author):

Over the past 50 years, the two most significant improvements of grain yield have been achieved by deploying semidwarfism and exploitation of heterosis in indica rice breeding programs. However, the molecular mechanisms underlying rice heterosis still remains unclear. In this manuscript, the authors reported that they have developed a new QTL mapping method GradedPool-Seq to rapidly map QTLs by whole-genome sequencing graded pool-samples from F2 progeny with bulked segregants analysis. They also used this new method to rapidly map and clone the heterotic genes in super-hybrid rice variety. The topic of this manuscript is interest for researchers follow trends to dissect a heterotic QTL/gene and understanding of molecular mechanism underlying heterosis. Most of my comments are relatively minor, but a number of aspects of the manuscript are not clear and need to be improved.

1, based on the widely different plant phenotypes and t their frequency distribution, F2 populations could be classified into three types: Grade 1 (i.e., the highest bulk), Grade 2 (i.e., middle bulk), and Grade 3 (i.e., the lowest bulk). It is easy to understand that the SNP variations were not related to the heterotic phenotype, it would present 50% reference reads and 50% alternate reads in all three bulks, whereas the related SNP would have great distinction of the ratio of reference reads to alternate reads between Grade 1 and Grade 3. In Fig.1b, the related SNP in Grade 1 was associated only with higher ratio of reference reads to alternate reads, it is also possible that the related SNP in Grade 1 was associated with lower ratio of reference reads to alternate reads. Thus, the authors should include this additional analysis when they mapped the heterosis-related genes associated with three or more graded groups based on the measured phenotypic values of contrasting phenotypes.

2, in the previous studies, the authors have reported that they developed the interval mapping method and mapped several heterozygous locus which played an important role for yield-related traits, including GW3p6. Using this new method GradedPool-Seq, is it possible to dissect the new heterosis-related QTL/genes associated with grain production?

3, the GW3p6 allele is same as the previously reported OsMADS1lgy3 allele. This alternatively spliced protein OsMADS1lgy3 has been shown to be associated with the increased grain size and grain yield. In addition, the previous studies have demonstrated that the introduction of the OsMADS1lgy3 allele into indica hybrid rice resulted in increases in both grain length and grain weight, to increase grain yield by a mean of 7.1%. Thus, the part of functional analysis and yield improvement of OsMADS1GW3p6 can be removed.

4, according to Supplementary Table 2, the OsMADS1GW3p6 allele can only be detected in 51 hybrid combinations among 1,328 varieties of hybrid rice, particularly in the first generation of hybrid rice (three-line system). This result suggests that the OsMADS1GW3p6 allele is not commonly used in hybrid rice, and it doesn't play an important role to contribute the rice heterosis. How can we use this new method GradedPool-Seq to dissect OsMADS1GW3p6 or other heterosis-related genes associated with grain production?

5, previous studies have demonstrated that the alternatively spliced allele is a semi-dominant allele with respect to grain length, grain weight and grain yield per plant. In this manuscript, the author showed that the near isogenic line (NIL-FH::GW3p6) had a large increase in yield compared with FH, but was still less production slightly to F1. How to explain these different type of observations in F1?

Reviewer #2 (Remarks to the Author):

In this manuscript, Wang CS et al describe novel methodology that allows QTL mapping of F2

populations by sequencing of bulked pools sampled across the distribution of trait phenotypes. This approach was applied to lines derived from F1 crosses between hybrid line parents and enabled the identification of regions that contribute to the observed heterosis between the parents for grain/yield traits. This approach enabled fine mapping of a previously identified QTL, GW3p6, to an interval of 5.9kb. They validated that the allele in GZ63-4S at candidate gene in this interval, OsMADS1, contributes to heterosis between GZ63-4S and FH676 supported by increased grain yield in NILs containing this allele. All of these results are significant and of broad interest to the community.

However, the authors have based their alignment and mapping on the Nipponbare build 4.0 from 2009. Yet, Kawahara et al (2013) released an improved build of Nipponbare, IRGSP 1.0 (Rice, 6:4 doi: 10.1186/1939-8433-6-4); this build of the Nipponbare genome is now the preferred build. In order for their results to be more easily accessible, coordinates presented in the results should be remapped to the updated build.

Additionally, several high-quality rice genome builds for indica type genomes are available that have qualities on par with that of the temperate japonica type Nipponbare:

NCBI Assembly IDs:
R498.Genome.version1 Shuhui498 (PacBio 120x) 2017/05/04
Rice_IR8_v1.7 IR 8 (PacBio 70x) 2016/12/06
ZS97RS2 Zhenshan 97 (PacBio 120x) 2018/10/23
MH63RS2 Minghui 63 (PacBio 120x) 2018/10/25
ASM386521v1 93-11 (PacBio 116x) 2018/12/06

Since both parental lines in this study are indica types, some comparison at osMADS1 locus to the most relevant indica build(s) is warranted. Since GZ63-4S has Minghui 63 in its parentage (and MH63 has IR8 in its), the most appropriate genomes for comparison are MH63 and IR8. This comparison would extend the results beyond the description of its occurrence in the resequenced genomes from the authors in their prior studies.

The manuscript needs significant editing to improve the English. I suggest that a native English speaker undertake this task or that they avail of a service provider. The edits were too numerous for me to list them all.

Some examples:
Line 21 change to: … contribution to solving the food crisis.
Line 22 change to: …heterosis for grain yield is mainly …
Line 29 change to: …sequencing of graded pool-samples …
Line 34 Use “that” NOT “which”
Line 40 change to: … demonstrated that heterotic genes …
Line 43 change to: … without the need to construct …

Reviewer #3 (Remarks to the Author):

Review: Dissecting a heterotic gene through GradedPool-1 Seq mapping informs a new
2 rice-improvement strategy, by Wang et al.

This manuscript describes the development and utilization of a new quantitative trait loci (QTL) mapping approach (GradedPool-Seq;GPS) using bulked progeny (segregant) next-generation sequencing of rice F2 population. Following phenotype of a population derived from F1 hybrid, which was showing superior phenotype for growth traits over the mid-parent phenotype (heterosis), the

plants are grouped to low, mid and high values. Next, DNA from representative of each group are subjected to whole genome sequencing for identification of enriched alleles in the highest pool. Using GPS methodology authors managed to obtain mapping to a resolution of app. 400Kbp for grain weight QTL. This was further fine-mapped using larger F2 population derived from a RIL down to a resolution of a known single gene of rice, i.e. GW3p6 (OsMADS1), that confer a significant increase of app. 8% for grain weight and length. These partially dominant effects were also confirmed in nearly isogenic lines for this allele originating from maternal parent, and effects were also observed in genome edited mutated plants for this gene that showed malformed seeds. Functional analysis of the identified alternative spliced variants implicate the C-termini function, which is supported by previous studies on this gene. Furthermore, allele mining of the rice gene pool, or Pan Genome, indicate that this grain-weight increasing allele is found only in small portion of the rice breeding material. These results led authors to suggest GPS as a novel method for dissecting heterosis to its elusive genetic components and utilize this directly on breeding plant material with less need to construct non-predictive hybrids.

Overall, the presentation of the data is very clear and straightforward. The authors combine both empirical data and simulations on the same dataset to highlight the power of their method. Now, there are several limitations that the authors fail to mention despite their knowledge in heterosis in general, and that of grain yield particularly. Grain yield is of course the golden grail of heterosis, and the more interesting trait that different models are attempting to resolve. The GPS method, at least the way presented, is compatible for single plant traits. This is true for F2 population composed of individuals that are both genotyped and phenotypes, however traits as grain yield requires groups of plants or plots to obtain phenotypic value, and so is the need to find QxE interactions. Perhaps the authors could try is to mention this limitation and test the compatibility of the GPS for F2:F3 families, for example, in which genotype is conducted in the F2 population and the grain yield phenotype in F3. Although this dilutes the allelic effect by half or more since quarter and half of selfed heterozygous plant are homozygous and heterozygous for the allele of interest. At least this is how it used to be done in advanced backcross method by Tanksley, for example, who tried to solve the use of introgression directly to breeding. Currently, the GPS is good as any other QTL mapping approach for continuous traits, other than yield that requires plots.

Second point with regard to grain yield heterosis that is overlooked, or at least I could not find any mentioning even in supplementary data, is that the GPS is not compatible with the overdominant model for heterosis, which does appear in the introductory part of the manuscript (row 64). In case the gene contributing to hybrid vigor is overdominant, i.e. heterozygous genetic value surplus both homozygous for two alleles, then how would GPS identify such locus? If it would not, then at least this should have been discussed.

The results section begins with describing the GPS methodology. It states the selection of 100-150 individuals from each bulk. It is indeed useful and relevant to have simulations to understand better the power of the GPS to identify QTL (supplementary Fig. 5) For the sake of clarity the number of plants in bulk be better presented as percentage of the F2 population and not a number of individuals. This is true for presenting the information in the text, as well as in the figures and supplementary table 1. One naive question in that regard is what is the proportion of plants collected for DNA extraction? If one needs to phenotype all, extract DNA (from individuals) for half or more, then what is the cost-effectiveness in the whole process? These consideration needs some more explanation. In addition, it would be useful to understand in these simulations the needed QTL effect in order for it to be identified.

In the last part of the results authors present pyramiding of the OsMADS1_GW3p6 with another QTL for a grain yield component, i.e. PN3q23. I found the presentation of the results somewhat confusing. First, it is obvious that there seems to be epistatic interactions or diminishing effects between the two QTL and that the carrier of both QTL does not show a significant effect of the single QTL. In general,

without having a detailed table, rather than a bar plot and pie chart that confuses the reader it is impossible to judge the added value of this pyramiding. By eyeball of bars and large STE of the double introgression it seems that having one or two QTL does not seem to make a significant difference, but I may be wrong. Again, a detailed table will clarify this.

Minor comments:
Abstract:
Second sentence in abstract is a bit redundant (heterosis appears twice). So are the last two sentences in review, with redundant "demonstrated", and grammatical mistake (need and not needs).

Introduction:
¬ rice hybrid varieties are not necessarily heterozygous in any locus (row 50).
¬ > rows 60 -66 are too lengthy for a single sentence
Results
¬ Row 135—to compute and not computer

The authors refer the reader to a link that 1) written in Chinese, and 2) cannot be displayed. This does not allow to asses the GPS software as part of this review.

To conclude, this manuscript is divided between to two parts, i.e. the QTL mapping by GBS, and the proof for the functionality of the gene on a quantitative trait. Both are impressive is breadth, but none of them is fully novel, i.e. there are previous similar QTLseq approaches that take almost similar approaches, and the functional analysis of the GW3p6 was shown previously by map based cloning efforts. The only perhaps novelty is the finding of a less used allele in the maternal gene pool that implicate this for rice breeding, and the success in pyramiding two QTL to achieve 14% increase in single plant grain yield although the implications in the field still require additional proof. In addition, it would strengthen the manuscript to add some discussion on the limitations of the GPS (see above- single plant traits, dominant genes,etc.) rather than paint it all positive but this could be a matter of personal preference.

Responses to referees for the manuscript of NCOMMS-19-00043A:

Reviewer #1

1, Based on the widely different plant phenotypes and their frequency distribution, F2 populations could be classified into three types: Grade 1 (i.e., the highest bulk), Grade 2 (i.e., middle bulk), and Grade 3 (i.e., the lowest bulk). It is easy to understand that the SNP variations were not related to the heterotic phenotype, it would present 50% reference reads and 50% alternate reads in all three bulks, whereas the related SNP would have great distinction of the ratio of reference reads to alternate reads between Grade 1 and Grade 3. In Fig.1b, the related SNP in Grade 1 was associated only with higher ratio of reference reads to alternate reads, it is also possible that the related SNP in Grade 1 was associated with lower ratio of reference reads to alternate reads. Thus, the authors should include this additional analysis when they mapped the heterosis-related genes associated with three or more graded groups based on the measured phenotypic values of contrasting phenotypes.

Yes, it is a very good comment. In the previous Figure 1b, we only presented one situation that the related SNP in Grade 1 was associated with higher ratio of reference reads to alternative reads, whereas we did not show the other situation that the related SNP may have lower ratio of reference reads to alternate reads. We have corrected the Figure 1 in the revision. The newly modified Figure 1 includes the analysis of two situations when we mapped the heterotic genes associated with multiple groups based on the measured phenotypic values of contrasting phenotypes. We also added the description in the text (Line113-115).

2, in the previous studies, the authors have reported that they developed the interval mapping method and mapped several heterozygous locus which played an important role for yield-related traits, including GW3p6. Using this new method GradedPool-Seq, is it possible to dissect the new heterosis-related QTL/genes associated with grain production?

Yes, it is possible to dissect new heterosis-related genes association with grain production using

the GradedPool-Seq method. In the results of Figure 2b, the new heterosis-related QTLs are also shown. On chromosome 6, there is an unknown heterosis-related QTL with high effect value, which need further verification.

In our previous studies, Composite Interval Mapping (CIM) method is still powerful and has many advantages in QTL mapping. For example, it can improve the efficiency and the accuracy of mapping by controlling background genetic effects to a large extent. However, CIM is also time-consuming in rice hybrid breeding because each line in the large population needs to be genotyped. Therefore, we developed the new GPS method to rapidly map heterozygous loci. Compared to the CIM method, the GPS method may have higher efficiency when extremely large sample size is used. Thus, it is convenient to dissect new heterosis-related genes association with grain production using the GradedPool-Seq method.

3, the GW3p6 allele is same as the previously reported OsMADS1lgy3 allele. This alternatively spliced protein OsMADS1lgy3 has been shown to be associated with the increased grain size and grain yield. In addition, the previous studies have demonstrated that the introduction of the OsMADS1lgy3 allele into indica hybrid rice resulted in increases in both grain length and grain weight, to increase grain yield by a mean of 7.1%. Thus, the part of functional analysis and yield improvement of OsMADS1GW3p6 can be removed.

We did notice that *OsMADS1*^{lgy3} can increase grain yield in a restorer line 9311. The mode of inheritance of the *OsMADS1*^{lgy3} is semi-dominant, indicating an important role of *lgy3* allele in hybrid rice. In our study, we directly cloned the *OsMADS1*^{GW3p6} from the male-sterile line Guangzhan63-4S (a commonly used parent in hybrid rice breeding) by GPS, and identified the *OsMADS1*^{GW3p6} to be a grain weight-related heterosis gene. We also introduced the *GW3p6* allele from male-sterile line into the restore line Fuhui676 to demonstrate a significant yield-improvement of NIL-FH::*GW3p6* over the Fuhui676. This could be a complementary result to the previous reports. In addition, we applied transient expression assay of promoter activity to prove that the change of 1000-grain weight is not related to the promoter, and the results of gene editing in the C Domain of *OsMADS1* proved that the alternatively spliced protein *OsMADS1*^{GW3p6} was associated with the increased grain size and grain yield.

4, according to Supplementary Table 2, the OsMADS1GW3p6 allele can only be detected in

51 hybrid combinations among 1,328 varieties of hybrid rice, particularly in the first generation of hybrid rice (three-line system). This result suggests that the OsMADS1GW3p6 allele is not commonly used in hybrid rice, and it doesn't play an important role to contribute the rice heterosis. How can we use this new method GradedPool-Seq to dissect OsMADS1GW3p6 or other heterosis-related genes associated with grain production?

Our Supplementary Table 2 (Supplementary Table 3 currently) showed that approximately 4% hybrid rice contained the *OsMADS1*^{GW3p6}, because most accessions of hybrid rice in our collection belonged to hybrid rice of three-line system. Generally speaking, the proportion of *OsMADS1*^{GW3p6} is low in the hybrid rice of three-line system, and modest in the hybrid rice of two-line system. We found that *OsMADS1*^{GW3p6} may come from *japonica* rice -- from the pan-genome data of *OsMADS1*^{GW3p6}, we can find that *OsMADS1*^{GW3p6} is an allele widely existing in tropical *japonica* rice, but most hybrid rice varieties have *indica* background resulting the relatively low proportion of *OsMADS1*^{GW3p6} in hybrid rice. Therefore, by dissecting the function of *OsMADS1*^{GW3p6}, we can make better use of the yield-increasing effect of *OsMADS1*^{GW3p6}. Considering that *OsMADS1*^{GW3p6} showed a significant improvement to grain weight, it will have great potential in hybrid rice breeding, and will play an important role on contributing to the rice heterosis.

The GPS method improves the efficiency of identifying heterosis genes greatly. In more hybrid rice varieties, we can focus on more heterosis traits, and find more heterosis genes by GPS. A whole set of pipeline is provided in the article, and three major sections is also shown in Supplementary Figure 1. After that, introducing heterosis genes to restore line or male-sterile line and selection of hybrid combinations purposefully will be beneficial to hybrid rice breeding.

5, previous studies have demonstrated that the alternatively spliced allele is a semi-dominant allele with respect to grain length, grain weight and grain yield per plant. In this manuscript, the author showed that the near isogenic line (NIL-FH::GW3p6) had a large increase in yield compared with FH, but was still less production slightly to F1. How to explain these different type of observations in F1?

Although we have demonstrated that the NIL-FH::*GW3p6* has a significant yield-improvement over the Fuhui676 or NIL-FH::*GW3p6/*Fuhui676, we have to mention that the high

grain-production of the hybrid rice GLY-676 (F1) is resulted from genetic effects of multiple alleles. Detailed explanations are as following:

Firstly, the grain yield of F_1 generation is controlled by multiple genes. Although the near isogenic line (NIL-FH::*GW3p6*) had a large increase in grain production compared with FH, it is still far from enough to rely on the introduction of only one gene, even if the gene production effect of the $OsMADS1^{GW3p6}$ is powerful. That's why we continue to find new heterosis genes from male-sterile line (for example, another heterosis related gene *PN3q23* underlying panicle number). When pyramiding two or more heterosis-related genes, the gap between the grain production of F_1 and near isogenic line will be smaller.

Secondly, numerous minor genes also may play a role in heterosis. It is hard to map and character the minor QTL genes, but the effect of a largr number of minor genes is one of the reasons why the grain yield of NIL-FH:*GW3p6* is less than F_1.

Thirdly, the over-dominance effects of certain heterozygous genes and epistatic effects among different genes may also contribute to grain yield of F_1.

We added these discussions in the revision (Line375-376).

Reviewer #2

Thank you for your recognition of our work, we want to make a big step-forward on rapidly dissecting complex traits regarding to heterosis for rice breeding. Therefore, a good and accessible result will help breeders to do hybrid rice breeding more efficiently.

1.However, the authors have based their alignment and mapping on the Nipponbare build 4.0 from 2009. Yet, Kawahara et al (2013) released an improved build of Nipponbare, IRGSP 1.0 (Rice, 6:4 doi: 10.1186/1939-8433-6-4); this build of the Nipponbare genome is now the preferred build. In order for their results to be more easily accessible, coordinates presented in the results should be remapped to the updated build.

Thanks for the comment. The mapping and alignment works in our lab were carried out with the reference of IRGSP 4.0 since 2009. In the revision, we have performed additional analyses by using the build of Nipponbare, IRGSP1.0 (Rice,6:4 doi:10.1186/1939-8433-6-4) for re-mapping the heterosis-related genes. In the results of four traits, we remapped the heterosis genes based on

Os-Nipponbare-Reference-IRGSP-1.0. The results by IRGSP 4.0 and those by Os-Nipponbare-Reference-IRGSP-1.0 were almost the same. We also remapped the 1000-grain weight genes in GLY-676 based on the improved IRGSP 1.0. The new results were presented in Supplemental Figure 6. The remap results based on Os-Nipponbare-Reference-IRGSP-1.0 are also nearly the same with those from IRGSP 4.0. We added the comparisons as suggested in the main text (Lines 190-193).

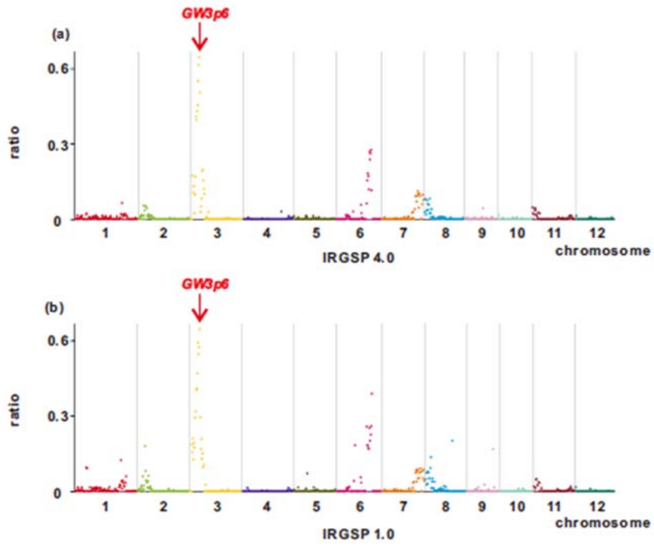

2.Additionally, several high-quality rice genome builds for indica type genomes are available that have qualities on par with that of the temperate japonica type Nipponbare:

NCBI Assembly IDs:

R498.Genome.version1 Shuhui498 (PacBio 120x) 2017/05/04

Rice_IR8_v1.7 IR 8 (PacBio 70x) 2016/12/06

ZS97RS2 Zhenshan 97 (PacBio 120x) 2018/10/23

MH63RS2 Minghui 63 (PacBio 120x) 2018/10/25

ASM386521v1 93-11 (PacBio 116x) 2018/12/06

Since both parental lines in this study are indica types, some comparison at osMADS1 locus to the most relevant indica build(s) is warranted. Since GZ63-4S has Minghui 63 in its parentage (and MH63 has IR8 in its), the most appropriate genomes for comparison are MH63 and IR8. This comparison would extend the results beyond the description of its

occurrence in the resequenced genomes from the authors in their prior studies.

Guangzhan63-4S is a widely planted photoperiod-temperature sensitive male sterile line, and its background is *indica*. We also remap the heterosis genes by BWA and GATK based on *indica* MH63 genome. The comparison results are showed as figure in Supplemental Figure 6. The results of GPS are almost identical among different genomes. These new results were added in the revision and the papers for all the new high quality genome sequences were cited.

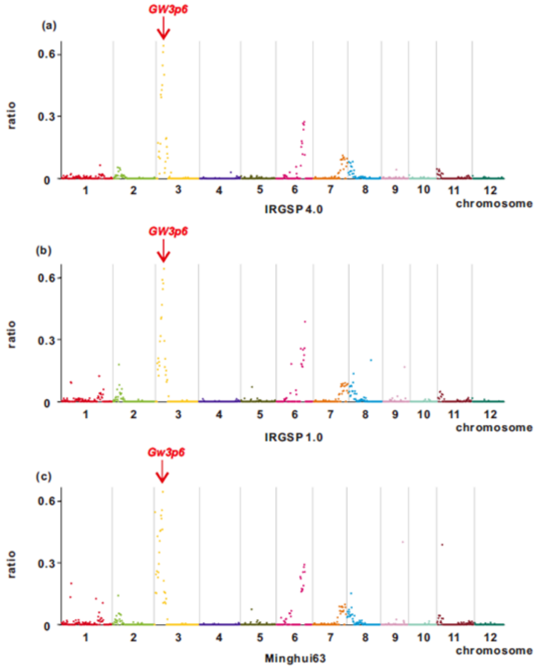

3. The manuscript needs significant editing to improve the English. I suggest that a native English speaker undertake this task or that they avail of a service provider. The edits were too numerous for me to list them all.

Some examples:

Line 21 change to: … contribution to solving the food crisis.

Line 22 change to: …heterosis for grain yield is mainly …

Line 29 change to: …sequencing of graded pool-samples …

Line 34 Use “that” NOT “which”

Line 40 change to: … demonstrated that heterotic genes …

Line 43 change to: … without the need to construct …

We are very honored that you have wasted a lot of energy in providing us language guidance. We have revised the manuscript word for word, and some grammatical errors and inappropriate expressions are avoided as far as possible. At the same time, we also invite service providers to further polish, hoping to make the manuscript more accessible and understandable.

Review#3

1.Overall, the presentation of the data is very clear and straightforward. The authors combine both empirical data and simulations on the same dataset to highlight the power of their method. Now, there are several limitations that the authors fail to mention despite their knowledge in heterosis in general, and that of grain yield particularly. Grain yield is of course the golden grail of heterosis, and the more interesting trait that different models are attempting to resolve. The GPS method, at least the way presented, is compatible for single plant traits. This is true for F2 population composed of individuals that are both genotyped and phenotypes, however traits as grain yield requires groups of plants or plots to obtain phenotypic value, and so is the need to find QxE interactions. Perhaps the authors could try is to mention this limitation and test the compatibility of the GPS for F2:F3 families, for example, in which genotype is conducted in the F2 population and the grain yield phenotype in F3. Although this dilutes the allelic effect by half or more since quarter and half of selfed heterozygous plant are homozygous and heterozygous for the allele of interest. At least this is how it used to be done in advanced backcross method by Tanksley, for example, who tried to solve the use of introgression directly to breeding. Currently, the GPS is good as any other QTL mapping approach for continuous traits, other than yield that requires plots.

We appreciate reviewer's recognition of our work, and we agree with the kind reviewer that our new GPS approach is as good as any other QTL mapping approach currently. However, our methods have low power in finding QxE interactions and other limitations, which were added in the discussion sections of the revision (Line 347-350). Thanks!

2. Second point with regard to grain yield heterosis that is overlooked, or at least I could not find any mentioning even in supplementary data, is that the GPS is not compatible with the

overdominant model for heterosis, which does appear in the introductory part of the manuscript (row 64). In case the gene contributing to hybrid vigor is overdominant, i.e. heterozygous genetic value surplus both homozygous for two alleles, then how would GPS identify such locus? If it would not, then at least this should have been discussed.

Yes! The GPS may not work very well with the overdominant loci. To identify QTLs through the software GPS, the SNPs related to the agronomic traits need have a great distinction of reference reads to alternative reads. However, for the overdominant loci, there may be a great distinction of heterozygous genotypes to homozygous genotypes, but less great distinctions of reference reads to alternative reads. We added the statement in the revision (Line350-356).

3.The results section begins with describing the GPS methodology. It states the selection of 100-150 individuals from each bulk. It is indeed useful and relevant to have simulations to understand better the power of the GPS to identify QTL (supplementary Fig. 5) For the sake of clarity the number of plants in bulk be better presented as percentage of the F2 population and not a number of individuals. This is true for presenting the information in the text, as well as in the figures and supplementary table 1. One naive question in that regard is what is the proportion of plants collected for DNA extraction? If one needs to phenotype all, extract DNA (from individuals) for half or more, then what is the cost-effectiveness in the whole process? These consideration needs some more explanation. In addition, it would be useful to understand in these simulations the needed QTL effect in order for it to be identified.

We agree. We should present the number of plants in bulk as percentage, but not the number of individuals. We added a new column of percentage in Supplementary Table 1 as suggested. In the text, we also added the description in the text (Lines 109-110 and Lines 613-614).

For BSA and whole-genome sequence of bulked DNA, we added the detailed description in methods section (lines 603-610 and lines 617-628). In F_2 populations, we usually phenotype all F_2 individuals for a certain trait, and the F_2 population sizes in this work are not very large (typically, ~500 individuals), it's easy to phenotype all. According to the phenotypic values, we divided F_2

individuals into several pools. The pool size and the number of pools are based on population size and phenotypic differences. After that, the genomic DNA were extracted from the mixed equal mass leaf tissue of F_2 individuals in each pool. The equal mass fresh leaf tissue (~0.05g) of each individual was mixed in a mortar, and then the genomic DNA of them was extracted for further sequencing. The cost of extracting DNA in equal mass fresh is economical.

For the cost-effectiveness aspect in the whole process of GPS, we think it's very economical than other fine-mapping methods. Applying GPS doesn't require to genotype all individuals, which is time-saving and labor-saving (especially, we will not need to perform sequencing library construction for each line). More details in the whole process were added in the text (lines 334-346).

For QTL effect, we agree the reviewer #3's comment. We added the description on QTL effects in Supplementary Note 1 (Lines 92-93).

4.In the last part of the results authors present pyramiding of the OsMADS1_GW3p6 with another QTL for a grain yield component, i.e. PN3q23. I found the presentation of the results somewhat confusing. First, it is obvious that there seems to be epistatic interactions or diminishing effects between the two QTL and that the carrier of both QTL does not show a significant effect of the single QTL. In general, without having a detailed table, rather than a bar plot and pie chart that confuses the reader it is impossible to judge the added value of this pyramiding. By eyeball of bars and large STE of the double introgression it seems that having one or two QTL does not seem to make a significant difference, but I may be wrong. Again, a detailed table will clarify this.

We have provided a detailed table to show the yield-increasing effect of the plants harboring the two heterosis related genes. The detailed table were presented as Table 2 in manuscript (see below). The number of the plants containing *PN3q23* and *GW3p6* is small, which caused eyeball of bars and large STE of the double introgression. We only have a small number seeds of GLY-676, and we chose nearly the same number of NIL plants, although the number of NIL plants is enough. We have used more NIL plants to calculate the yield increasing effect, and more detailed data about yield per plant is available in source data. We initially thought the bar plot and pie chart were more intuitive, but judging the added value of genes' pyramiding is impossible. From the

results of Table 2, the grain yield of plants carrying *GW3p6* and *PN3q23* is significantly higher than that of FH, as well as NIL-FH::*GW3p6*. In addition, we still provide the pie chart as Figure 5b. Benefiting from the results of Table 2, it's easy to understand the pie chart. The calculation method of heterotic contribution rate is described in the methods (Line726-733). The yield-increasing effect of the plants containing *GW3p6* and *PN3q23* proved the genes with incomplete dominance from maternal parent played a significant role in heterosis. The pie chart of heterosis contribution could show a small number heterosis genes explained the majority of heterosis effects.

Table 2. Grain yield per plant of FH, the plants containing *GW3p6* and *PN3q23*, NIL-FH::*GW3p6*, and GLY676

(I) Genotype	No. of plants	Grain yield per plant (g)[a,b]	(J) Genotype	Mean differences (I-J)	Standard error	Significance (*p-value*)[c]
FH	70	42.644±2.796	GLY-676	-14.234	1.222	4.319×10^{-14}
			NIL-FH::*GW3p6*	-3.971	0.726	7.267×10^{-7}
			GW3p6+PN3q23	-6.323	0.726	7.894×10^{-14}
NIL-FH::*GW3p6*	70	46.615±2.853	GLY-676	-10.263	1.222	1.082×10^{-13}
			FH	3.971	0.726	7.267×10^{-7}
			GW3p6+PN3q23	-2.352	0.726	7.479×10^{-3}
GW3p6+PN3q23	70	48.968±6.330	GLY-676	-7.911	1.222	3.662×10^{-14}
			FH	6.323	0.726	7.894×10^{-14}
			NIL-FH::*GW3p6*	2.352	0.726	7.479×10^{-3}
GLY-676	15	56.878±3.888	FH	14.234	1.222	4.319×10^{-14}
			NIL-FH::*GW3p6*	10.263	1.222	1.082×10^{-13}
			GW3p6+PN3q23	7.911	1.222	3.662×10^{-14}

[a] All data are given as means±SD.

[b] Data for the grain yield per plant are based on a field experiment using a randomized complete block design.

[c] *P* value produced by the Tukey's HSD.

In addition, in the process we pyramiding another gene *PN3q23* underlying panicle number, we found the plants carrying *PN3q23* had greater number of panicles than FH and NIL-FH::*GW3p6*, besides the yield-increasing effect. The detailed information was provided as Supplementary Table 2.

Supplementary Table 2. Panicle number of FH, the plants containing *GW3p6* and *PN3q23*, NIL-FH::*GW3p6*, and GLY676

(I) Genotype	No. of plants	Panicle number per plant (n) [a,b]	(J) Genotype	Mean differences (I-J)	Standard error	Significance (*p-value*)[c]
FH	70	7.200±1.566	GLY-676	-7.667	0.478	3.653×10^{-14}
			NIL-FH::*GW3p6*	0.014	0.284	1.000
			GW3p6+PN3q23	-1.114	0.284	6.755×10^{-4}
NIL-FH::*GW3p6*	70	7.186±1.544	GLY-676	-7.681	0.478	3.653×10^{-14}
			FH	-0.014	0.284	1.000
			GW3p6+PN3q23	-1.129	0.284	5.568×10^{-4}
GW3p6+PN3q23	70	8.314±1.975	GLY-676	-6.552	0.478	3.653×10^{-14}
			FH	1.114	0.284	6.755×10^{-4}
			NIL-FH::*GW3p6*	1.129	0.284	5.568×10^{-4}
GLY-676	15	14.867±1.246	FH	7.667	0.478	3.653×10^{-14}
			NIL-FH::*GW3p6*	7.681	0.478	3.653×10^{-14}
			GW3p6+PN3q23	6.552	0.478	3.653×10^{-14}

[a] All data are given as means±SD.
[b] Data for the grain yield per plant are based on a field experiment using a randomized complete block design. The panicle number is counted one week before harvesting.
[c] *P* value produced by the Tukey's HSD.

5.Minor comments:

Abstract:

Second sentence in abstract is a bit redundant (heterosis appears twice). So are the last two sentences in review, with redundant "demonstrated", and grammatical mistake (need and not needs).

Introduction:

Ø rice hybrid varieties are not necessarily heterozygous in any locus (row 50).

Ø > rows 60 -66 are too lengthy for a single sentence

Results

Ø Row 135—to compute and not computer

We are very grateful to you for pointing out the mistakes in language so carefully. We have corrected it as suggested.

6.The authors refer the reader to a link that 1) written in Chinese, and 2) cannot be displayed. This does not allow to asses the GPS software as part of this review.

We have optimized our pipeline of GPS to be smoother and simpler. We also provided a single compressed zip file containing the software with a detailed readme.txt. In addition, we have corrected the mistakes raised by reviewer 3.

7.To conclude, this manuscript is divided between to two parts, i.e. the QTL mapping by GBS, and the proof for the functionality of the gene on a quantitative trait. Both are impressive is breadth, but none of them is fully novel, i.e. there are previous similar QTLseq approaches that take almost similar approaches, and the functional analysis of the GW3p6 was shown previously by map based cloning efforts. The only perhaps novelty is the finding of a less used allele in the maternal gene pool that implicate this for rice breeding, and the success in pyramiding two QTL to achieve 14% increase in single plant grain yield although the implications in the field still require additional proof. In addition, it would strengthen the manuscript to add some discussion on the limitations of the GPS (see above- single plant traits, dominant genes,etc.) rather than paint it all positive but this could be a matter of personal preference.

Thanks for the comments. The QTL-seq approaches combine BSA (bulked-segregant analysis) with genome-wide sequencing to identify QTL associated with target traits. Although the GPS approach has some similarities to QTL-seq, they have some differences in terms of algorithm and results. We also repeated the data of QTL-seq method, and the supplementary figure 4 showed that the GPS had a higher resolution. We hope the highly efficient GPS method can dramatically accelerate crop improvement in a cost-effective manner.

In addition, we added some discussion on the limitation of the GPS as suggested, it’s helpful for improving the level of the article. Thanks again.

REVIEWERS' COMMENTS:

Reviewer #1 (Remarks to the Author):

In this revised version, the authors have added additional data and answered the questions which I addressed, now it is accepted for publication.

Reviewer #2 (Remarks to the Author):

GPS provides a useful addition to the breeder's toolkit, and your analyses have contributed further to development of hybrid rice.

Thank you for considering all of my comments and addressing them. It is nice to see the comparison of mapping between different Nipponbare builds and to see results for mapping to the indica type Minghui 63. Further, I appreciate your effort improving the grammar, spelling and writing. There are a few places, especially in the added text, where there are minor problems.
For example on line 32, it should be "inbred" not "inbreed".

Reviewer #3 (Remarks to the Author):

The introduction of next generation sequencing (NGS) in the past few years have led to the development of quantitative trait loci (QTL) discovery by establishing and phenotyping a segregating population and selecting individuals with high and low values for the trait of interest, which are characterized for differences in allele frequencies. One most prominent pipeline, termed QTLseq, was described by Takagi et al. (2013) and since then was widely used in several crop plants for many traits. The other main computational tools to evaluate statistical significance of QTL from NGS-BSA was proposed by Magwene et al. (2011) [Magwene, P.M., J.H. Willis, and J.K. Kelly. 2011. The statistics of bulk segregant analysis using next generation sequencing. PLOS Comput. Biol.], in which G statistic is calculated for each SNP based on the observed and expected allele depths and the value is smoothed by considering relative distance of neighboring SNPs. Of course, these are not the only studies that deal with this QTL mapping approach (only recently came a new paper by Mansfeld and Grumet in Plant Genome, QTLseqr: An R Package for Bulk Segregant Analysis with Next-Generation Sequencing) but they do give a perspective on the state-of-the-art in this field. Now, this study by Wang et al. is presenting a new analysis termed GradedPool-Seq for dissecting QTL in rice for grain weight traits using F2 population, and resequencing three groups of bulked individuals, each is app. 20-30% of the whole phenotyped population. Already in the introductory part the authors fail to present, for example, the Magwene (2011) work, and in general it seems that there is too much avoidance from what has been done in this field. Instead, the comparison of the new GPS method is conducted to mapping of qualitative trait mutants, and with regard to QTL it is compared only to the QTL-seq only. The authors show that the GPS has app. 5X more resolution, i.e. from 2M bp to app. 400 Kbp. In fact, I find this difference not very significant; having a 5X resolution would both requires follow-up mapping population to achieve a single gene resolution. And Indeed, this is shown in this work with an heterozygous recombinant inbred lines used for finer mapping of the QTL after this was mapped in F2 population. To summarize, I don't find this difference between QTL-seq and GPS significant enough with regard to change in the breeding or gene cloning procedures. Moreover, considering other studies in the field, e.g. Magwene et al. 2011 and Mansfeld and Grumet (2019), it seems that this study is of greater interest to those interested in rice genetics.

Reviewer #1 (Remarks to the Author):

In this revised version, the authors have added additional data and answered the questions which I addressed, now it is accepted for publication.

Thank you for your comments.

Reviewer #2 (Remarks to the Author):

GPS provides a useful addition to the breeder's toolkit, and your analyses have contributed further to development of hybrid rice.

Thank you for considering all of my comments and addressing them. It is nice to see the comparison of mapping between different Nipponbare builds and to see results for mapping to the indica type Minghui 63. Further, I appreciate your effort improving the grammar, spelling and writing. There are a few places, especially in the added text, where there are minor problems.
For example on line 32, it should be "inbred" not "inbreed".

Thank you for checking our manuscript carefully. And We are grateful for your positive comments. We revised the manuscript word for word to correct grammar and check spelling again.

Reviewer #3 (Remarks to the Author):

The introduction of next generation sequencing (NGS) in the past few years have led to the development of quantitative trait loci (QTL) discovery by establishing and phenotyping a segregating population and selecting individuals with high and low values for the trait of interest, which are characterized for differences in allele frequencies. One most prominent pipeline, termed QTLseq, was described by Takagi et al. (2013) and since then was widely used in several crop plants for many traits. The other main computational tools to evaluate statistical significance of QTL from NGS-BSA was proposed by Magwene et al. (2011) [Magwene, P.M., J.H. Willis, and J.K. Kelly. 2011. The statistics of bulk segregant analysis using next generation sequencing. PLOS Comput. Biol.], in which G statistic is calculated for each SNP based on the observed and expected allele depths and the value is smoothed by considering relative distance of neighboring SNPs. Of course, these are not the only studies that deal with this QTL mapping approach (only recently came a new paper by Mansfeld and Grumet in Plant Genome, QTLseqr: An R Package for Bulk Segregant Analysis with Next-Generation Sequencing) but they do give a perspective on the state-of-the-art in this field. Now, this study by Wang et al. is presenting a new analysis termed GradedPool-Seq for dissecting QTL in rice for grain weight traits using F2 population, and resequencing three groups of bulked individuals, each is app. 20-30% of the whole phenotyped population. Already in the introductory part the authors fail to present, for example, the Magwene (2011) work, and in general it seems that there is too much avoidance from what has been done in this field. Instead, the comparison of the new GPS method is conducted to mapping of qualitative trait mutants, and with regard to QTL it is compared only to the QTL-seq only. The authors show that the GPS has app. 5X more resolution, i.e. from 2M bp to app. 400 Kbp. In fact, I find this difference not very significant;

having a 5X resolution would both requires follow-up mapping population to achieve a single gene resolution. And Indeed, this is shown in this work with an heterozygous recombinant inbred lines used for finer mapping of the QTL after this was mapped in F2 population. To summarize, I don't find this difference between QTL-seq and GPS significant enough with regard to change in the breeding or gene cloning procedures. Moreover, considering other studies in the field, e.g. Magwene et al. 2011 and Mansfeld and Grumet (2019), it seems that this study is of greater interest to those interested in rice genetics.

First of all, we appreciate the reviewer's comments. In our introduction section, we emphatically introduce several QTL mapping methods, but not statistical algorithms. Now we cite Mansfeld and Grumet's paper in Introduction section to enrich our manuscript. As the reviewer said, the methods we mentioned were not the only studies that deal with QTL mapping. Our GPS method with Ridit analysis will be a very good complement to the QTL mapping work and rice breeding. The reasons why we chose QTL-seq method as the comparison: (1) QTL-seq can work in F_2 population (2) the similarity between QTL-seq and GPS method in experimental design (3) QTL-seq can map QTLs (4) QTL-seq is a popular method in QTL mapping work currently. Therefore, the higher resolution of GPS than that of QTL-seq method will be convincing in QTL mapping.

The high resolution in QTL mapping will help to accelerate the progress of fine-scale mapping and breeding. Of course, GPS cannot achieve a single gene resolution. But applying GPS method in gene cloning and breeding will be cost-effective and time-saving.
